# Generalized nuclear localization of retroelement transcripts

**DOI:** 10.1186/s13100-022-00287-x

**Published:** 2022-12-02

**Authors:** Simanti Das, Amanda E. Jones, John M. Abrams

**Affiliations:** Department of Cell Biology, University of Texas Southwestern Medical Center, Dallas, TX 75390 USA

**Keywords:** Retroelements, RNA, Nuclear localization, RNA-sequencing, LINE-1, Development, Retrotransposition

## Abstract

**Background:**

LINE-1s, Alus and SVAs are the only retrotransposition competent elements in humans. Their mobilization followed by insertional mutagenesis is often linked to disease. Apart from these rare integration events, accumulation of retrotransposition intermediates in the cytoplasm is potentially pathogenic due to induction of inflammatory response pathways. Although the retrotransposition of LINE-1 and Alu retroelements has been studied in considerable detail, there are mixed observations about the localization of their RNAs.

**Results:**

We undertook a comprehensive and unbiased approach to analyze retroelement RNA localization using common cell lines and publicly available datasets containing RNA-sequencing data from subcellular fractions. Using our customized analytic pipeline, we compared localization patterns of RNAs transcribed from retroelements and single-copy protein coding genes. Our results demonstrate a generalized characteristic pattern of retroelement RNA nuclear localization that is conserved across retroelement classes as well as evolutionarily young and ancient elements. Preferential nuclear enrichment of retroelement transcripts was consistently observed in cell lines, in vivo and across species. Moreover, retroelement RNA localization patterns were dynamic and subject to change during development, as seen in zebrafish embryos.

**Conclusion:**

The pronounced nuclear localization of transcripts arising from ancient as well as de novo transcribed retroelements suggests that these transcripts are retained in the nucleus as opposed to being re-imported to the nucleus or degraded in the cytoplasm. This raises the possibility that there is adaptive value associated with this localization pattern to the host, the retroelements or possibly both.

**Supplementary Information:**

The online version contains supplementary material available at 10.1186/s13100-022-00287-x.

## Introduction

Retroelements are genomic sequences that have the potential to mobilize (i.e., retrotranspose) to a different genomic location via an RNA intermediate. Although retroelement sequences are abundant in the genome, the majority have mutations, truncations and rearrangements that render them inactive and incapable of retrotransposition. Structurally, retroelements can be divided into two main categories, Long Terminal Repeat (LTR) and non-LTR retrotransposons. As the name suggests, LTR retrotransposons have long terminal repeats on either side of a region encoding retrovirus-like *gag* and *pol* genes. Endogenous retroviruses (ERVs) are an example of LTR retroelements that make up nearly 8% of the human genome [1]. LTRs can no longer mobilize in humans, but some elements do retain limited protein coding ability [2]. Among non-LTR retroelements in humans, Long Interspersed Nuclear Elements (LINEs) are the most abundant and make up ~ 17% of the genome. Although there are ~ 600,000 LINE-1 (L1) derived sequences in the genome, there are only 100–150 full length and retrotransposition competent L1 elements. Functional L1 copies are characterized by the presence of an intact 5′ untranslated region (UTR) and the ability to encode functional ORF1p and ORF2p proteins [3–5]. Short Interspersed Nuclear Elements (SINEs) such as Alus and SVAs are also non-LTR retroelements [1]. Alus and SVA retroelements lack any protein coding capacity and rely on the LINE-1 machinery for retrotransposition [6, 7].

As the only autonomously active human retroelement, LINE-1 s have been extensively studied. A typical L1 retrotransposition cycle starts with transcription of LINE-1 DNA into mRNA followed by its translation into two proteins: ORF1p, which has nucleic acid chaperone activity and ORF2p which functions as an endonuclease (EN) and reverse transcriptase (RT) [8–11]. Next, a ribonucleoprotein (RNP) complex consisting of L1 RNA and ORF proteins re-enters the nucleus where ORF2p encoded endonuclease cleaves target DNA, and target primed reverse transcription (TPRT) is initiated [12, 13]. The retrotransposition process is complete when reverse transcribed LINE-1 DNA is integrated at the target site and DNA breaks are repaired through endogenous cellular pathways [14].

L1 mobilization is potentially mutagenic and novel L1 insertions have been identified in Hemophilia A, Duchenne Muscular Dystrophy and Neurofibromatosis Type I patients, with approximately 1% of sporadic genetic diseases thought to result from de novo L1 integrations [15]. In somatic tissues, L1 mobilization is strongly associated with neoplastic disease and is detectable in more than 50% of human cancers, often associated with p53 loss [16–19]. L1 mobilization has also been shown to trigger large scale genomic rearrangements commonly seen in cancer genomes [20, 21].

In addition to the direct effects of L1 insertions, L1 intermediates including RNA, RNA:DNA hybrid structures, and L1 encoded proteins have also been linked to disease. L1 RNAs can potentially activate inflammatory pathways and cause diseases, including amyotrophic lateral sclerosis (ALS) and Aicardi–Goutières syndrome [22–24]. Sense and antisense transcription from retroelements produces complementary RNAs that can form double-stranded RNAs (dsRNAs) which, through viral mimicry, can trigger Toll-Like receptor (TLR) and Tumor Necrosis Factor- α (TNF-α) mediated immune response [25]. Furthermore, increased RT in the cytoplasm can lead to reverse transcription of retroelement RNAs in the cytosol and the resulting dsDNA and RNA:DNA hybrids can cause activation of the cGAS/STING inflammatory pathway, while nuclear endonuclease expression can create double stranded DNA breaks [10, 25–29]. Retroelement RNAs have also been reported to be dysregulated in certain cancers although it is unclear if these RNAs play a role in transformation [19, 30, 31]. Much of the pathogenic potential for L1 intermediates is, therefore, determined by subcellular localization.

Full length, polyadenylated LINE-1 transcripts were first detected in cytoplasmic RNA fractions of Ntera2D1 teratocarcinoma cells by Northern blot [32]. Since then, several reports inspecting endogenous elements or engineered constructs have produced conflicting observations about localization of retroelement transcripts within the cell. Endogenous L1 RNAs as well as MS2-tagged L1 RNAs, colocalize with cytoplasmic stress granules and autophagosomes suggesting autophagy may inhibit retrotransposition [33–36]. Similarly, exogenously expressed Alu transcripts have also been reported to be predominantly localized in cytoplasmic autophagosomes [35]. In mice, L1 RNA is abundant in the nucleus of embryonic stem cells (mESCs) and pre-implantation embryos [37]. As a further complexity, studies conducted with ORFeus and L1rp derived L1 mRNAs suggest that cell cycle may impact localization [38]. L1 and Alu RNA, as part of C_0_T-1 repeat RNA, was found to be highly enriched in the nucleus in several human cell lines [39]. When we attempted to stably knockdown endogenous human L1 RNAs to investigate their role in oncogenesis, we found that L1 RNAs were considerably enriched in the nucleus. Therefore, to better define patterns and determinants of retroelement transcript localization, we undertook a comprehensive analysis using common cell lines and publicly available sequencing datasets.

We provide empirical and bioinformatic evidence suggesting that endogenous LINE-1 as well as other retroelement RNAs, with or without protein coding potential, accumulate in the nucleus. Moreover, preferential localization of retroelement transcripts to the nucleus is a generalizable phenomenon and is observed not only in cell lines but in vivo and across species*.* Finally, we observe developmentally triggered shifts in retroelement RNA localization in zebrafish embryos, which is likely driven by the onset of zygotic transcription of these elements.

## Results

### LINE-1 RNAs are enriched in the nucleus in human cells

**Fig. 1:**
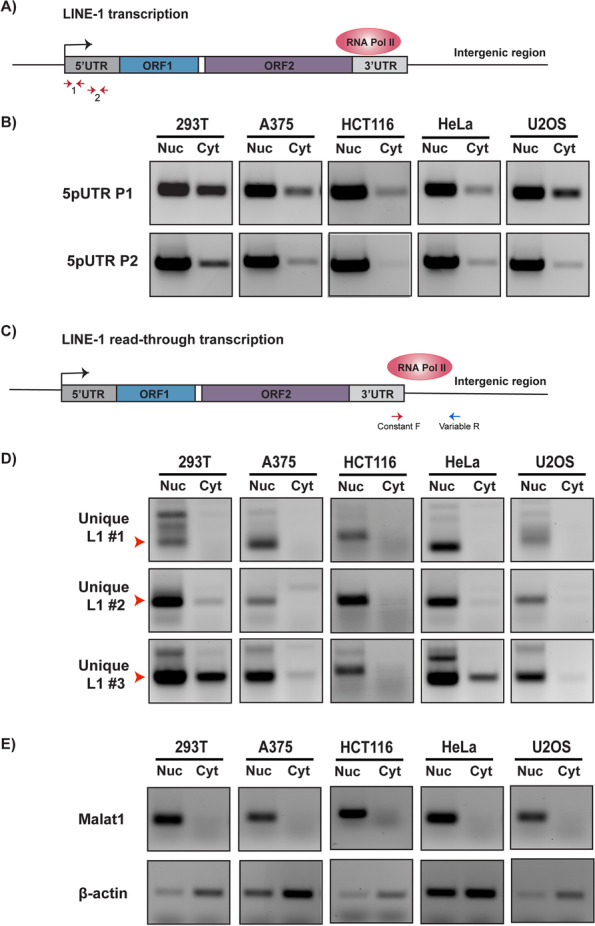
Retrotransposition competent LINE-1 RNA preferentially localizes to the nucleus in human cell lines. Transcripts expressed from retrotransposition competent L1-Hs were assayed by RT-PCR in nuclear (Nuc) and cytoplasmic (Cyt) RNA fractions from 293T, A375, HCT116, HeLa and U2OS cell lines. **a** Schematic depiction of LINE-1 transcription. Transcription is initiated by RNA Pol II at the internal promoter in the 5’UTR. Red arrows marked 1 and 2 indicate the position of primer sets 5pUTR P1 and 5pUTR P2 respectively. **b** Full-length LINE-1 transcripts are highly enriched in the nucleus. The two primer sets used, 5pUTR P1 and 5pUTR P2, indicated by the two sets of red arrows in (A), detect all expressed L1-Hs with an intact 5’UTR. **c** Schematic depiction of read-through transcription from a LINE-1 coding sequence. Due to a weak 3p UTR polyA signal, RNA Pol II transcription can continue beyond the non-unique L1 3’UTR into downstream unique intergenic regions. Red and blue arrows indicate the position of the constant forward and variable reverse primers that were used to uniquely detect read-through transcription from individual, identifiable L1-Hs elements. **d** Transcripts from three distinct and uniquely identifiable L1-Hs also show nuclear enrichment of their transcripts in all the cell lines used in our assay. Red arrow heads indicate the expected amplicon size. Unique L1 #1 and #2 refer to specific L1-Hs elements on chromosome 7, whereas unique L1 #3 refers to a target L1-Hs element on chromosome 13 (see Additional File 15 for genomic coordinates of unique L1 copies). **e** Malat1, a nuclear RNA, and spliced β-actin transcript, which primarily localizes to the cytoplasm, serve as fractionation controls

We examined nuclear and cytoplasmic distribution of endogenous LINE-1 retroelement transcripts in cellular fractions from five well characterized human cell lines (293T, A375, HCT116, HeLa and U2OS). Although nearly 17% of the human genome is composed of LINE-1 sequences, most of those elements are inactive. Therefore, expression of LINE-1 human specific (L1-Hs), the only autonomous and active retroelement present in the human genome, was measured using specific primers (indicated by the red arrows in Fig. 1A) targeting the L1-Hs 5′ UTR. As seen in Fig. 1B, L1-Hs transcripts were enriched in the nuclear fraction of all studied cell lines. Importantly, known nuclear (Malat1) and cytoplasmic (spliced β-actin) enriched RNAs were appropriately localized, indicating a clean cellular fractionation (Fig. 1E). Next, we sought to quantify transcripts from uniquely identifiable copies of L1-Hs elements, since the data in Fig. 1B represents consolidated signals from many different L1-Hs copies. Read-through transcription (illustrated in Fig. 1C), wherein RNA Polymerase II (RNA Pol II) transcription continues beyond the L1-Hs polyadenylation signal into unique downstream sequences, is common for many L1-Hs copies [40, 41]. We designed primer sets (see Methods) specific for three distinct L1-Hs copies by pairing a conserved L1-Hs 3′ UTR primer (red arrow) with a variable primer (blue arrow) complementary to unique downstream sequence (see Additional File 6). As seen in Fig. 1D, we observed that transcripts expressed from each of these uniquely identifiable L1-Hs were similarly enriched in the nucleus across all cell lines. Therefore, when assayed either in bulk or as unique, identifiable copies, L1-Hs transcripts were enriched in the nucleus.

### LINE, LTR and SINE transcripts are highly nuclear across human cell lines

To ask whether these findings extend to other retroelements and cell lines, we analyzed publicly available datasets from Production ENCODE Project (PRJNA30709, [42–44]). From this database, we proceeded with the analysis of RNA-sequencing data from paired nuclear and cytoplasmic fractions of six cell lines (A549, HUVEC, IMR90, K569, MCF7 and SK-N-SH). These six cell lines were selected based on several criteria (see Methods for details). Most relevant here, we required long paired-end reads to ensure superior mappability and at least two replicates, ensuring rigor and reproducibility.

To assess fractionation quality of these datasets, we calculated a measure of splicing completeness (1 = completely spliced, 0 = completely unspliced) called the Splicing Index (SI) for each sample (see Methods, [45]). As expected for well fractionated samples, most cytoplasmic transcripts are completely spliced (SI = 1), while many nuclear transcripts are partially spliced or unspliced (SI < 1) (Additional File 1A, compare red and green bars). We also measured standard sequencing quality metrics (Additional File 7A) including average per position sequencing coverage (see Methods, FastQC and Picard, Additional File 8A) and importantly all replicates of the six cell lines met our quality control metrics.

**Fig. 2:**
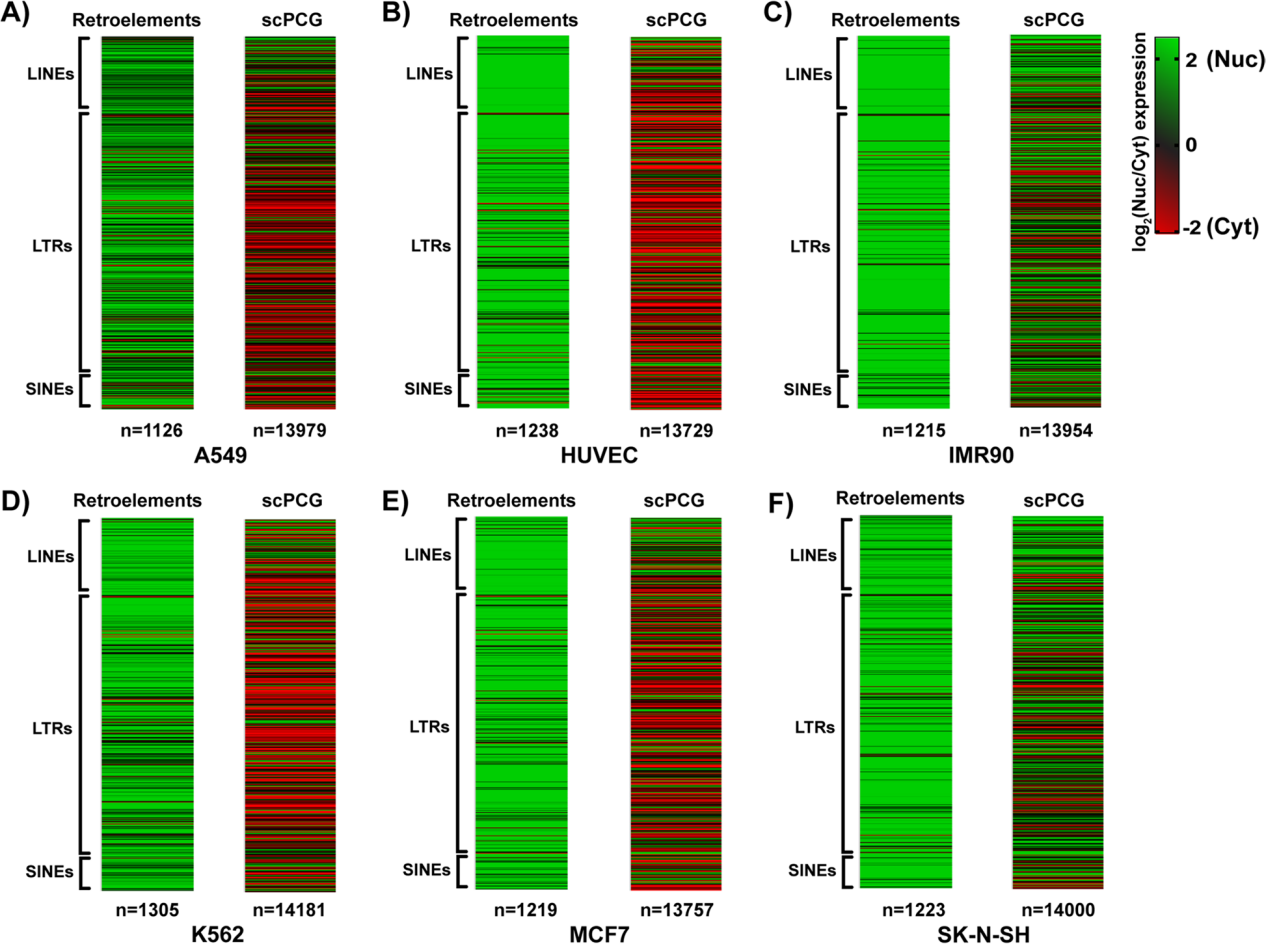
Transcripts from different classes of retroelements are enriched in the nucleus in human cell lines. RNA-sequencing data from nuclear (Nuc) and cytoplasmic (Cyt) fractions of A549, HUVEC, IMR90, K562, MCF7 and SK-N-SH cells was analyzed to study localization of retroelement RNAs. In (**a**) A549, (**b**) HUVEC, (**c**) IMR90, (**d**) K562, (**e**) MCF7 and (**f**) SK-N-SH cell lines, all classes of retroelements are highly enriched in the nucleus (heatmaps on the left for each pair) compared to single-copy protein coding genes (scPCGs) (heatmaps on the right for each pair). Each row in the heatmaps presents the log_2_ ratio of nuclear to cytoplasmic expression of a specific type of retroelement or a unique scPCG. Please note that ratios were calculated from consolidated signals summarizing the expression of individual copies of each element type. In all heatmaps, log_2_(Nuc/Cyt) values greater than 0 are represented as green and indicate nuclear localization, log_2_(Nuc/Cyt) values less than 0 are represented as red and indicate cytoplasmic localization whereas log_2_(Nuc/Cyt) values equal to 0 are represented as black and indicate no preferential localization. Retroelement and scPCG expression values were normalized to spike-in count (see Methods). Number of rows in each heatmap (n) is indicated. Please note that the group of scPCGs and individual retroelement types analyzed for each cell line may differ (see Methods for inclusion criteria)

Next, we analyzed each pair of cell fractions using an analytic pipeline customized for measuring retroelement expression (see Methods, [21]). Expression values for retroelements and single-copy protein coding genes (scPCGs) were obtained by normalizing raw read counts to the spike-in library (see Methods). Please note that, due to the repetitive nature of retroelement sequences, retroelement expression was calculated at the level of individual element types rather than individual genomic copies (see Methods for retroelement nomenclature used in this study). For each annotated retroelement type and scPCG, we calculated the log_2_ ratio of nuclear to cytoplasmic expression (log_2_(Nuc/Cyt)) and plotted the ratios as heatmaps (Fig. 2A-F compare left and right heatmaps for each pair). As shown in Fig. 2A-F, transcripts from LINE, LTR and SINE class retroelements show strong nuclear (green) localization patterns in all cell lines. In contrast, transcripts from single-copy protein coding genes were primarily enriched in the cytoplasm (red) or did not display preferential localization (black). Additionally, we normalized the raw read counts using DESeq2 default parameters (see Methods, [46]) and generated heatmaps of log_2_(Nuc/Cyt) ratios for retroelements and scPCGs. As seen in Additional File 1B-G, we observed similar transcript localization trends as presented in Fig. 2A-F. Therefore, patterns of retroelement RNA nuclear localization seen here were not dependent upon the normalization technique used. Taken together, our results show that diverse classes of retroelement RNAs (LINEs, LTRs, SINEs) are predominantly enriched in the nuclear compartment in human cell lines, unlike single-copy protein coding genes.

### Retroelement RNAs localize to the nucleus irrespective of protein-coding potential

Results in Fig. 2 suggest that diverse classes of retroelement RNAs are preferentially enriched in the nucleus. However, these classes contain a mixture of ancient and evolutionarily young families and subfamilies, some of which remain active (AluY) or maintain limited protein coding capacity (ERVK) [47, 48]. Therefore, we asked whether nuclear localized retroelement transcripts were specifically derived from active, functional elements as seen in Fig. 1D, or whether ancient retroelements were similarly nuclear localized.

**Fig. 3:**
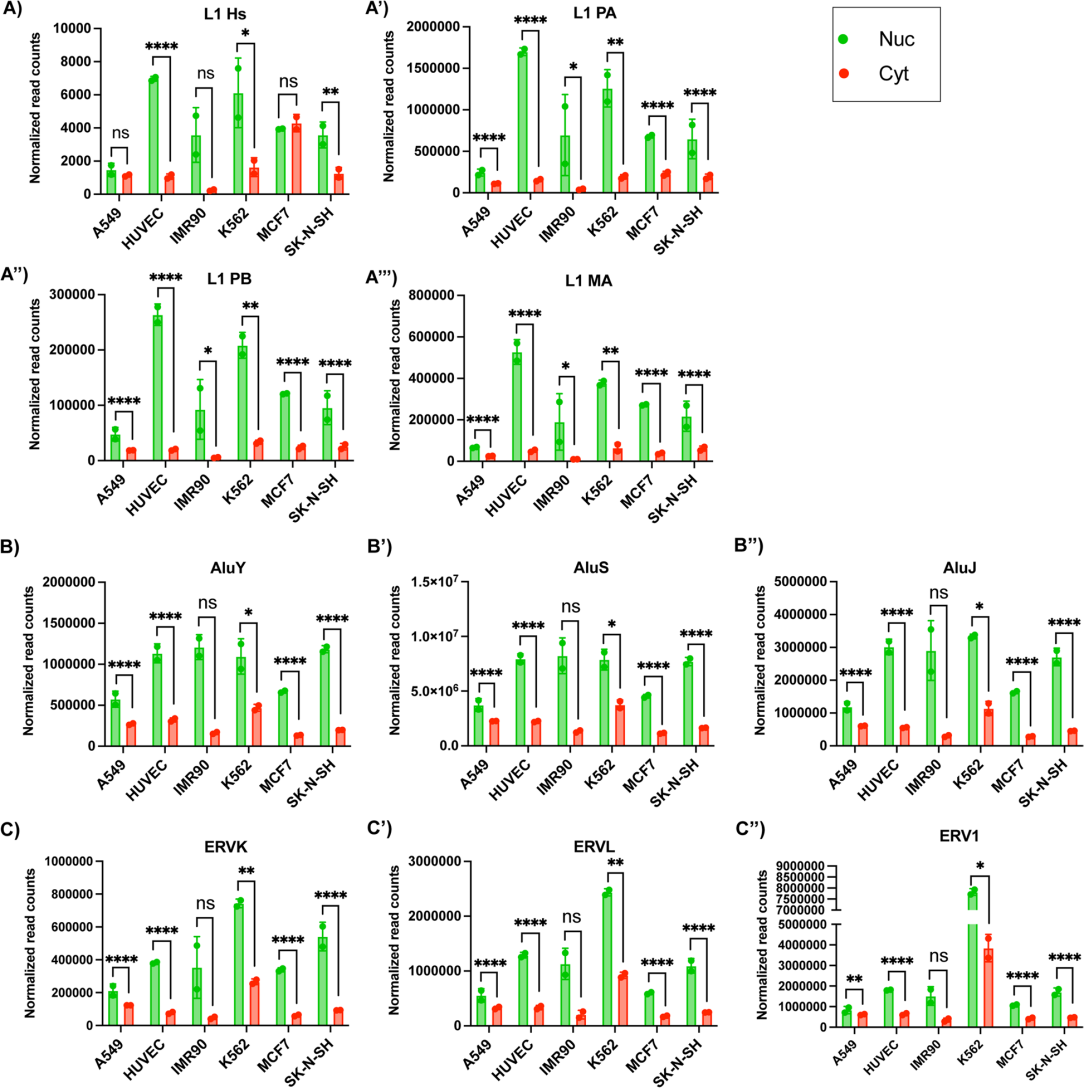
Evolutionarily young and old retroelements are enriched in the nucleus. **a** Transcripts from evolutionarily young and retrotransposition competent L1-HS are significantly nuclear enriched in multiple cell lines. (**A’-A”’**) Older LINE-1 subfamilies L1 PA and L1 PB found across primates and L1 MA found across mammals produce transcripts that are also significantly nuclear localized in all cell lines tested. (**B-B″**) Transcripts from elements in the three Alu subfamilies, AluY, AluS and AluJ, are present at significantly higher levels in the nucleus across all cell lines except in IMR90. (**C-C″**) Transcripts from elements belonging to the ERVK, ERVL and ERV1 families are significantly enriched in the nucleus as compared to the cytoplasm in all cell lines except in IMR90. For graphs in (**A-C″**), data points represent normalized read counts from two biological replicates and error bars indicate standard deviation. (ns = FDR > 0.05, * = FDR < 0.05, ** = FDR < 0.01, *** = FDR < 0.001, **** = FDR < 0.0001). FDR values reported here were direct outputs of EdgeR differential gene expression analysis

Reaffirming the results of our targeted experimental approach in Fig. 1B and D, we found that RNAs from L1-Hs elements were significantly enriched in the nucleus (FDR < 0.05) in three cell lines as shown in Fig. 3A. We also saw similar trends for L1-Hs in A549 and IMR90 but they were not significant at FDR < 0.05. Surprisingly, older LINE-1 subfamilies, including primate-specific L1 PA and L1 PB as well as mammal-specific L1 MA elements were also significantly enriched in the nucleus across all cell lines as shown in Fig. 3A’-A”’ (FDR < 0.05). Similarly, transcripts from both active and ancient Alu subfamilies also appeared to be enriched in the nuclear compartment across all cell lines (Fig. 3B-B″). Preferential nuclear localization was also seen in transcripts arising from ERVK, ERVL and ERV1 families in all cell lines (Fig. 3C-C″). These trends were significant (FDR < 0.05) in all studied cell lines except IMR90. These results demonstrate that transcripts from younger, retrotransposition competent elements as well as from ancient, non-coding elements show similar patterns of enrichment in the nucleus.

### Nuclear localization of retroelement transcripts occurs in vivo

Our results showed that human retroelement RNAs are generally localized to the nuclear compartment across many cell lines. Next, we wanted to investigate whether these localization patterns could also be observed in vivo*.* The publicly available dataset published by Price et al. (PRJNA595606, [49]) contains RNA-sequencing data from nuclear and cytoplasmic fractions of human pre-natal prefrontal cortex (PFC) and adult dorsolateral prefrontal cortex (DLPFC) tissue. An additional advantage of using these datasets was that for each tissue type, each sample was processed in parallel either by rRNA depletion (RiboZero) or by polyA selection (polyA) for sequencing library preparation. This gave us an opportunity to compare the effects, if any, of sequencing library preparation techniques on retroelement RNA localization. One sample of adult DLPFC (Br1113) was therefore excluded from our analysis because it lacked a RiboZero paired sample. All remaining samples met our quality metrics threshold for inclusion (Additional File 7B and 8B). Splicing indices were calculated for all samples, and as expected, RiboZero samples showed greater splicing completion in cytoplasmic fractions (Additional File 2A-B). The median splicing indices of the Nuc and Cyt fractions of polyA selected samples are nearly equal as seen in Additional File 3C-D and while Price et al. attributed this to overall pre-mRNA depletion due to polyA selection, notably we did not observe this for the polyA selected libraries prepared from human cell lines (Additional File 1A).

**Fig. 4:**
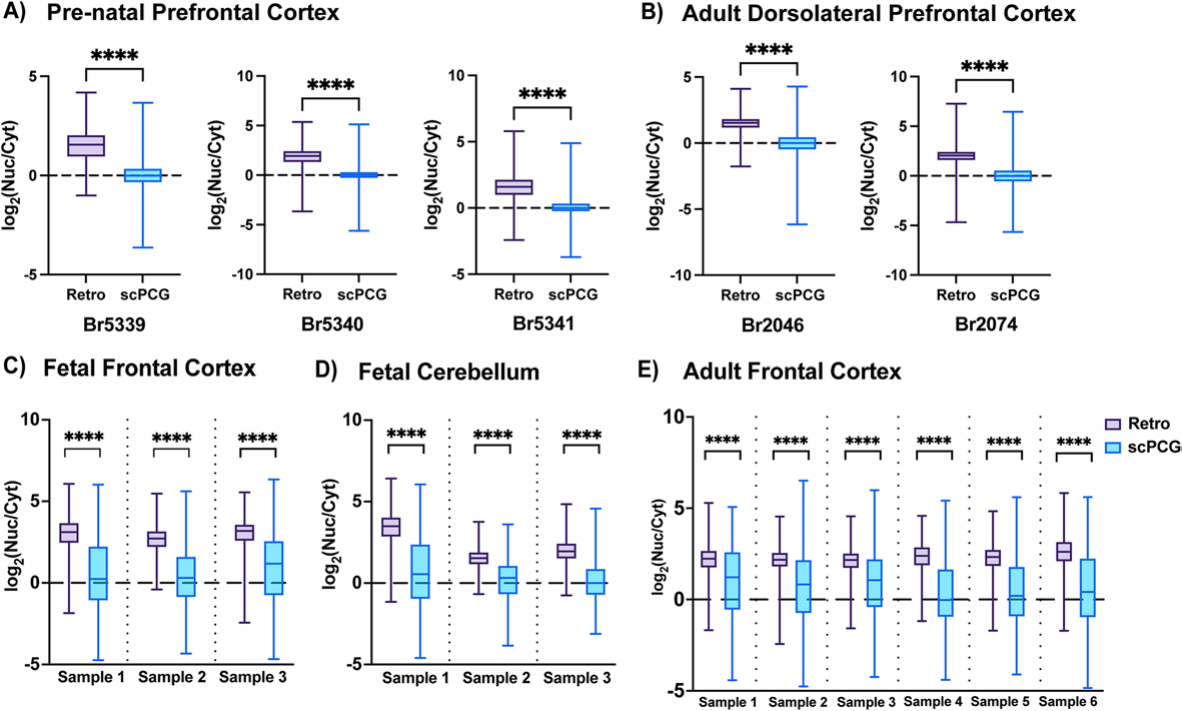
Retroelement RNAs localize to the nucleus in vivo. RNA-sequencing data from libraries prepared by rRNA depletion (RiboZero) from nuclear (Nuc) and cytoplasmic (Cyt) fractions of human brain tissue was analyzed to study retroelement RNA localization in vivo*.* Retroelement RNAs are significantly nuclear localized (MWU, *p* < 0.0001) unlike single-copy protein coding genes (scPCGs) in **a** Pre-natal prefrontal cortex (Br5339, Br5340, Br5341), **b** Adult dorsolateral prefrontal cortex (Br2046, Br2074) **c** Fetal frontal cortex (Sample 1–3), **d** Fetal cerebellum (Sample 1–3) and **e** Adult frontal cortex tissue (Sample 1–6). For each brain sample, the graphs in (**a-e**) plot log_2_(Nuc/Cyt) ratios plotted for all scPCGs and individual, annotated retroelement types (Retro) which had normalized read count > 10. Please note that ratios were calculated from consolidated signals summarizing expression of individual copies of each element type. The boundaries of the boxes denote 25th and 75th percentile and the whiskers mark the minimum and maximum values. The median log_2_(Nuc/Cyt) ratios are represented by the solid line at the center of the box. RNAs with log_2_(Nuc/Cyt) values greater than and less than 0 are considered nuclear and cytoplasmic transcripts respectively. Number of retroelements and scPCGs analyzed per sample are included in Additional File 9A and 9B

Next, we computed scPCG and retroelement expression values (see Methods) and calculated the log_2_(Nuc/Cyt) ratios. These ratios were then graphed in the form of box plots, and we considered RNAs with log_2_(Nuc/Cyt) ratios less than 0 as cytoplasmic and those greater than 0 as nuclear. Consistent with our observations in cell lines, we found that retroelement RNAs from both pre-natal PFC and adult human DLPFC exhibit an overall nuclear localization pattern whereas scPCG RNAs are relatively equally distributed in both nuclear and cytoplasmic fractions in RiboZero samples (Fig. 4A-B). The median log_2_(Nuc/Cyt) ratios for retroelements and scPCGs and Mann-Whitney U (MWU) *p*-values for all samples are listed in Additional File 9A. Interestingly, even though the localization trend of retroelement RNAs is consistent in polyA samples (Additional File 3A-B), the RiboZero datasets exhibit a more distinct difference than the polyA datasets.

To further validate our observations in in vivo samples, we similarly analyzed and graphed RNA-sequencing data from nuclear and cytoplasmic fractions of fetal (frontal cortex and cerebellum) and adult (frontal cortex) human brain tissue which was published by Zaghlool et al. (PRJNA434426, [50]). Sequencing libraries for this dataset were also prepared by rRNA depletion (RiboZero). Splicing indices for these samples are presented in Additional File 2C-E and quality metrics are included in Additional File 7C and 8C. Consistent with our previous observations, significant nuclear enrichment of retroelement transcripts was seen across all fetal and adult samples irrespective of the region of the brain from which tissue was sampled (Fig. 4C-E). The median log_2_(Nuc/Cyt) ratios for retroelements and scPCGs and Mann-Whitney U (MWU) *p*-values for all samples are listed in Additional File 9B. Although retroelement transcripts are relatively enriched in the nucleus we also note that substantial, albeit heterogeneous, levels remain detectable in the cytoplasm (see Additional File 10). Therefore, in vivo datasets produced by distinct groups, using different library preparation and sequencing methodologies, showed that nuclear localization of retroelement RNAs seen in cell lines normally occurs in tissues as well.

### Nuclear localization of retroelement RNAs in zebrafish embryos coincides with maternal to zygotic transition

**Fig. 5:**
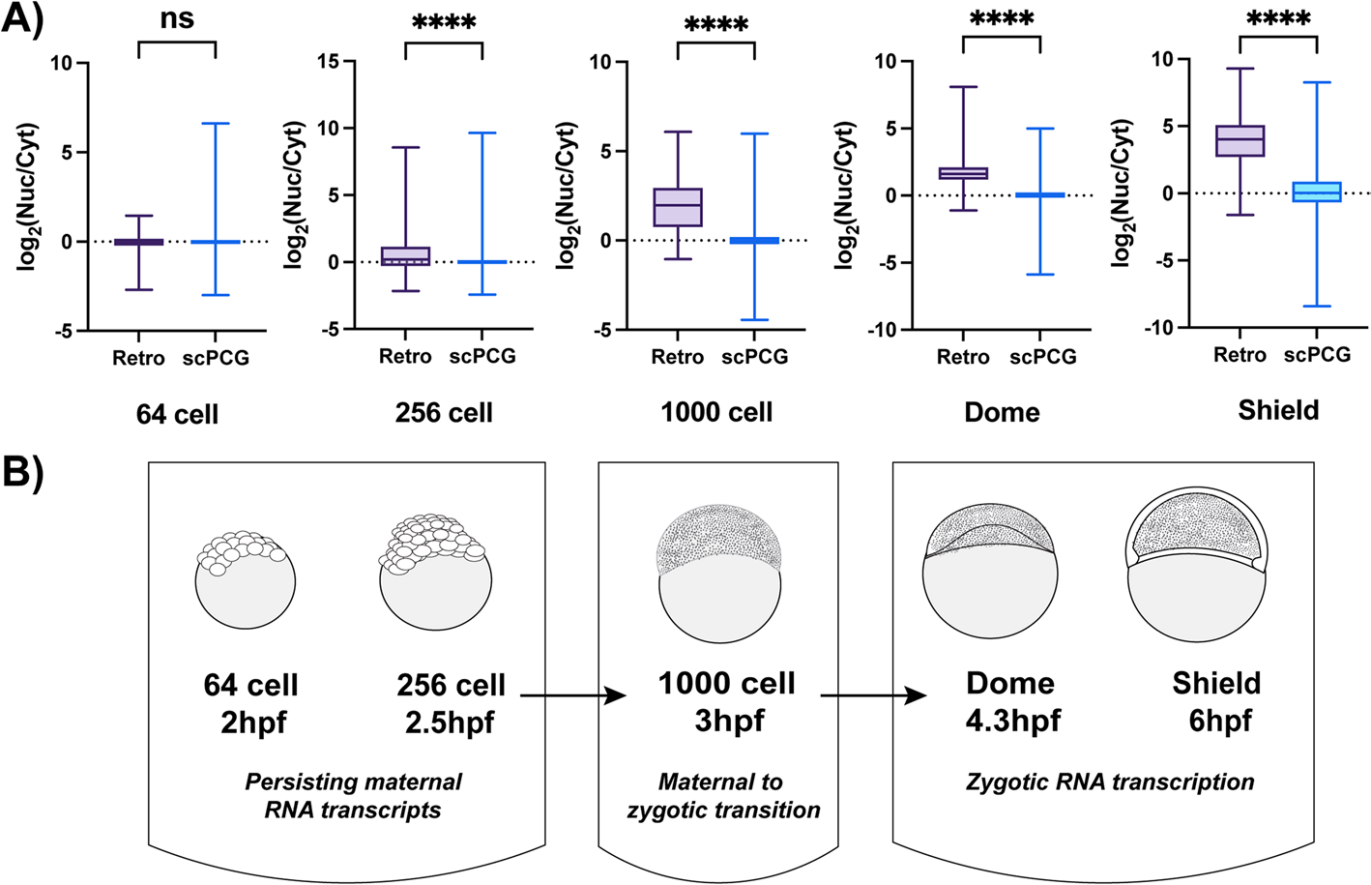
Patterns of retroelement RNA localization change during zebrafish embryo development. Retroelement transcript localization during zebrafish development was studied by analyzing RNA-sequencing data from libraries prepared by rRNA depletion (RiboZero) from nuclear (Nuc) and cytoplasmic (Cyt) fractions of 64 cell, 256 cell, 1000 cell, Dome and Shield stage zebrafish embryos. **a** Retroelement RNAs in zebrafish are relatively equally distributed in the nucleus and cytoplasm in 64 cell and 256 cell stages but are primarily nuclear localized starting at the 1000 cell stage (MWU, ns = *p* > 0.05, **** = *p* < 0.0001). RNA from single-copy protein coding genes (scPCGs) do not show preferential localization to any subcellular compartment during any stage of embryogenesis. The graphs show log_2_(Nuc/Cyt) ratios plotted for all scPCGs and individual, annotated retroelement types (Retro) which had normalized read count > 20 for each sample. Note that ratios were calculated from consolidated signals summarizing expression of individual copies of each element type. The boundaries of the boxes denote 25th and 75th percentile and the whiskers mark the minimum and maximum values. The median log_2_(Nuc/Cyt) ratios are represented by the line at the center of the box. Transcripts with log_2_(Nuc/Cyt) values greater than 0 are considered nuclear and those less than 0 are considered cytoplasmic. Number of retroelement types and scPCGs analyzed per sample are included in Additional File 9C. **b** Schematic diagram showing the developmental stages of zebrafish embryos included in this analysis and the overlap of maternal to zygotic transition (MZT) and zygotic genome activation (ZGA) with nuclear localization of retroelement RNAs (hpf = hours post fertilization)

To examine the dynamics of retroelement RNA localization in a developmental context and extend our study to other species, we next investigated RNA-sequencing data from zebrafish embryos. For this analysis, we obtained the datasets from Pillay et al. (PRJNA599208, [51]). These datasets consist of nuclear and cytoplasmic RNA-sequencing data from five different developmental stages of zebrafish embryos: 64 cell, 256 cell, 1000 cell, Dome and Shield stages. Note that sequencing libraries for these datasets were also prepared by rRNA depletion (RiboZero). Importantly, prior to the 1000 cell stage, zebrafish embryos are transcriptionally quiescent, with all detectable transcripts resulting from maternal loading during oogenesis (Fig. 5B). All samples met our threshold for quality metrics and were included in our analysis (see Additional File 7D and 8D). In addition, splicing indices for these samples are presented in Additional File 4A. Interestingly, in the 64 cell and 256 cell stage, the median splicing indices in both the nuclear and cytoplasmic fractions are close to 1 indicating that most transcripts present in the embryo are completely spliced. This likely reflects the presence of maternal RNAs synthesized and spliced during oogenesis and stored through the early stages of zygotic development [52]. Coinciding with the onset of zygotic transcription (1000 cell, Dome and Shield stages), the splicing index pattern returns to normal, with a cytoplasmic bias toward completely spliced transcripts.

As with previous analyses, we calculated the log_2_(Nuc/Cyt) ratio of expression values for retroelements and scPCGs where ratios greater than 0 and less than 0 indicate nuclear and cytoplasmic enrichment respectively. Notably, as seen in Fig. 5A, retroelement RNAs in the 64 cell and 256 cell stage exhibit localization patterns similar to scPCGS. However, during the 1000 cell stage retroelement RNA localization shifts, with RNAs in the Dome and Shield stage being almost entirely nuclear. This suggests that retroelement RNA localization patterns are dynamic and change during development. Although the difference between the median ratios for retroelements and scPCGs are statistically significant at the 256 cell stage, the effect size is quite small as compared to the 1000 cell, Dome and Shield stages (Additional File 9C). Interestingly, the shift to nuclear localization of retroelement RNAs starting at 1000 cell stage coincides with the maternal to zygotic transition (MZT) as seen in Fig. 5B [53, 54]. scPCG transcript localization was constant across developmental stages and, consistent with our previous observations, these transcripts were not enriched in either subcellular compartment. Importantly, increased nuclear localization patterns observed for retroelement RNAs during embryogenesis were not due to changes in the classes of retroelements expressed. As seen in Additional File 4B, although the number and expression of retroelements changed during zebrafish embryo development, the percentage of each class of retroelements expressed remained relatively constant.

## Discussion

To our knowledge, this study is the first to comprehensively examine retroelement RNA localization using next generation sequencing (NGS) data. Here we establish that, unlike single-copy protein coding genes, endogenous retroelement transcripts are preferentially localized to the nucleus. Importantly, these patterns were observed in vivo and in cell lines. Furthermore, we found this to be a generalizable phenomenon across species and across active and inactive families and subfamilies of retroelements belonging to the LINE, LTR and SINE classes. In addition, using zebrafish datasets, we found that patterns of nuclear localization for these RNAs coincide with the onset of zygotic transcription.

The datasets for our analyses were obtained from multiple sources, with different sample preparation, library preparation and sequencing methodologies. Nevertheless, we observed similar patterns of retroelement RNA nuclear localization pattern whether sequencing libraries were generated from polyA enriched or rRNA depleted samples. Therefore, mRNA purification methods were not responsible for producing these patterns. Likewise, datasets normalized using a spike-in library showed similar patterns of nuclear enrichment as those normalized using a standard TMM algorithm as implemented in DESeq2 [46]. Therefore, nuclear localization patterns seen here were also not driven by normalization methodologies.

Initially, we considered relevant RNA-sequencing datasets from mouse samples, published by Halpern et al. (PRJNA298634, [55]). Though fractionation quality was high for both samples (Additional File 5C-C′), we detected pronounced 5 prime degradation in all nuclear fractions (Additional File 5A-A’ and Additional File 8E). The same concern had also been reported by the authors [55] and, for this reason, we originally excluded these studies from our analyses. However, we recently revisited these datasets and, despite poor sample quality, nuclear localization of retroelement RNAs was clearly observed in mouse liver samples and a murine cell line (see Additional File 5B-B′). Therefore, even in less-than-optimal datasets we could still detect significant retroelement RNA nuclear localization, indicating that these patterns are robust.

We noted trends of nuclear enrichment of retroelement transcripts in the IMR90 cell line (Figs. 2C and 3), however the enrichment was not statistically significant in all cases. It is possible that the high level of PCR duplicates (87.41%) present in one of the replicates of the cytoplasmic fraction (Additional File 7A) impacted the accuracy of both the spike-in and DESeq2 normalization methods employed.

Recently, it was reported that incidental signal from L1 sequences nested within retained introns of protein coding genes could interfere with accurate quantification of L1 element expression patterns [56]. We therefore considered the possibility that signals from retroelements nested within retained introns could contribute to the pattern of nuclear enrichment that we observe. As shown in Additional Files 11 and 12, directed analyses of these nested LINE1 retroelements detect only low levels of intron derived sequences (compare expression of regions immediately upstream and downstream of nested and intergenic L1 sequences), and, importantly, the levels of intron derived sequences do not substantially differ in the cytoplasmic and nuclear fractions in sequencing libraries prepared from polyA enriched samples (compare cytoplasmic and nuclear fraction for each sample). As expected, intron derived sequences surrounding nested L1 sequences are relatively elevated in sequencing libraries prepared using Ribosomal RNA depletion, but, again, these levels do not differ in the cytoplasmic and nuclear fractions (Additional Files 13 and 14), suggesting that intron-embedded L1s do not bias our conclusions. Note that asterisks in Additional Files 11-14 mark the individual L1-Hs copies assayed by RT-PCR in Fig. 1D and the genomic coordinates for all L1-Hs copies displayed in these figures can be found in Additional File 15. Likewise, in zebrafish embryos, we find that signals from intron nested retroelements are similar between nuclear and cytoplasmic fractions and the patterns do not differ by developmental stage (Additional File 16, retroelement coordinates listed in Additional File 17). These combined results suggest that signals derived from retained introns do not significantly contribute to the nuclear localization pattern of retroelement transcripts.

The pronounced localization of retroelement RNAs to the nucleus, in contrast to scPCGs, suggests that retroelement and scPCG RNAs are subject to distinct regulatory mechanisms. Nuclear localization may result from nuclear re-entry, cytoplasmic degradation and/or nuclear retention of retroelement derived transcripts. Notably, full length L1 transcripts are actively imported to the nucleus following translation, a process dependent upon formation of the L1 RNP [57, 58]. However, although this process may contribute to the nuclear enrichment of full length L1 transcripts, our results show that RNAs from ancient non-coding LINE, SINE and LTR class retroelements are also highly enriched in the nucleus. Therefore, retroelement transcript localization patterns appear to be decoupled from autonomous retrotransposition. Cytoplasmic degradation of retroelement RNAs could also contribute to the localization patterns seen here. For example, degradation of retroelement transcripts by piRNAs and siRNAs in the cytoplasm has been demonstrated in both *Drosophila* and human cells and, although piRNAs are generally restricted to germline or embryonic cell types, retroelement targeting siRNAs remain active in somatic cells [59–62]. However, while cytoplasmic degradation may contribute to patterns of retroelement RNA nuclear localization, our results from zebrafish embryo RNA-seq data analysis suggest that retroelements transcribed de novo are nuclear localized whereas maternally sourced retroelement RNAs are not.

Taken together, our results raise the possibility that nuclear retention could be the primary mode of nuclear localization of retroelement transcripts. Indeed, some transposable element (TE) sequences (LINE2b, MIRb and MIRc) may drive the nuclear localization of long non-coding RNAs (lncRNAs) [63]. It has been proposed that these sequences can mediate nuclear retention of lncRNAs by interacting with nuclear proteins and hybridizing with complementary genomic DNA sequence [64]. Future experiments could identify the mechanism of nuclear retention and determine whether nuclear enrichment of retroelement RNAs is due to active retention or a failure to export.

Since successful retrotransposition events can lead to genomic instability and are potentially harmful to the host, retention of retroelement RNAs in the nucleus might be a host response to limit translation of retroelement derived proteins and thereby limit retrotransposition. Conversely, nuclear accumulation of retroelement RNAs may enable retroelements to evade the piRNA and siRNA host silencing machineries that are active in the cytoplasm [59–62]. Finally, this widespread, general pattern of nuclear localization may be an example of retroelement domestication, benefitting both the host and the transposon. For example, several groups have recently shown that retroelement transcripts can regulate gene expression and chromatin organization [37, 39, 65].

## Conclusion

Our study provides insight into a generalized property of retroelement RNAs that is broadly conserved across species. Future studies are needed to determine precisely how retroelement RNAs are selectively localized to the nucleus and determine whether sequence and/or structural determinants specify these patterns. Likewise, future studies interrogating retrotransposition rates, native gene expression and embryogenesis could shed light on the functional relevance of retroelement nuclear localization.

## Methods

### Cell culture

293T, A375, HeLa, U2OS and HCT116 cells were cultured in DMEM medium (Gibco, ThermoFisher Scientific) supplemented with 10% fetal bovine serum (FBS) (Peak Serum) and Antibiotic-Antimycotic (Gibco, ThermoFisher Scientific). The growth conditions of cell lines were kept constant at 37 °C, 5% CO_2_ and atmospheric O_2_.

### Cell fractionation and RNA extraction

For fractionation of cells, the published protocol from Cold Spring Harbor Laboratory was used [66]. Briefly, cells grown on 10 cm plates were collected by scraping them with PBS and washed 3X using PBS. The cells were then treated with Cell Disruption Buffer (10 mM KCl, 1.5 mM MgCl_2_, 20 mM TrisCl pH 7.5, 1 mM DTT) for 10mins followed by centrifugation to separate the nuclear and cytoplasmic fractions. RNA was extracted from nuclear fraction using TRIzol (Life Technologies) and from the cytoplasmic fraction using TRIzol LS (Life Technologies). DNase treatment was performed using the Turbo DNase kit (Invitrogen). Quantity and quality of RNA was determined using Nanodrop.

### cDNA preparation and RT-PCR assay

1 μg of RNA from each sample was used for cDNA synthesis reaction using iScript cDNA synthesis kit (Bio-Rad). For RT-PCR assays, GoTaq Master mix (Promega) was used with specific primers for each amplicon tested (see Additional File 6).

### Unique LINE-1 detection

Constant forward and variable reverse primers were designed to detect unique L1 readthrough transcription based on data from [41]. Two L1s on chr7 (111243517–111,249,546; 66,286,855–66,292,883) and one L1 on chr13 (31302315–31,308,344) were chosen based on their expression in multiple cell lines. Note that the coordinates correspond to the hg38 assembly. Primer sequences targeting bulk and unique L1s as well as Malat1 and β-actin are listed in Additional File 6.

### Data curation

For this study, we searched PubMed for bulk RNA-sequencing datasets (published prior to 2022) containing RNA-sequencing data from nuclear and cytoplasmic fractions of non-perturbed cells and tissues (human, mice, zebrafish). Cell line sequencing data from Production ENCODE project (PRJNA30709, [42–44]) was selected based on the following criteria: PolyA selection, paired-end sequencing, read length > =76nts and availability of two replicates. For the human brain dataset (PRJNA595606), adult DLPFC sample (Br1113) was excluded from our analysis as it did not have a matching RiboZero library. For the human brain dataset (PRJNA434426), only the tissue samples were analyzed (SHSY-5 cell line excluded).

### Quality control

As quality controls for the fractionations in this dataset, a metric called splicing index was defined [45].

$$Splicing\ index=\frac{No. of\ reads\ indicating\ splicing}{Total\ no. of\ informative\ reads\ }$$

The splicing index was calculated for each protein coding gene in the nuclear and cytoplasmic fractions of all samples analyzed. As RIN scores were not available for all RNA samples, we also assessed RNA integrity by computing the ratio of 5p to 3p read coverage for the 500 most highly expressed genes using Picard tools RNA metrics (http://broadinstitute.github.io/picard). Samples with a disparity of > 2.5 fold between 5p and 3p occupancy (normalized position 15 vs normalized position 85) were considered degraded and excluded from further analyses (Additional File 8A-E).

### RNA-seq data analysis

FastQ files from each dataset were pre-processed to remove adapter sequences, low quality 3′ and 5′ bases and poor-quality reads using Cutadapt v. 2.5 [67] and Prinseq v.0.20.4 [68]. Reads passing quality control filters were aligned to the UCSC annotations of the hg38 (human) and mm10 (mouse) genome assemblies or the Ensemble annotation of the GRCz11 (zebrafish) genome using STAR v. 2.7.8 (−-twopassMode Basic --outFilterMultimapScoreRange 2 --winAnchorMultimapNmax 1000 --outFilterMultimapNmax 10,000 --outFilterMismatchNmax 20) [69]. Duplicate reads were identified and marked using Picard v. 2.25.4 (http://broadinstitute.github.io/picard) and SAMtools v. 1.12 [70] and removed before further analysis. Note that aligned reads from the human brain dataset (PRJNA434426) were subsampled to match the number of reads in the smallest library due to substantial variation in number of reads remaining after deduplication and consistent with the methods employed by the authors [50]. The featureCounts program ( [71], http://subread.sourceforge.net), part of the Subread v2.0.2 package, was used to count unique reads that aligned to annotated scPCG transcripts. Note that for the mouse dataset (PRJNA298634), read counts for both fractions included only exonic reads in the last 500 bp from the 3′ end of the gene to adjust for 5p degradation in the nuclear fractions as indicated by the authors [55, 72]. The output counts tables were used as input for normalization using the default TMM algorithm in DESeq2 v. 2.11.40.6 as implemented in Galaxy [46]. Human cell line sequencing data from ENCODE project (PRJNA30709) was also normalized to the Illumina PhiX control library spiked in at 1% to each completed human library.

### Retroelement expression analysis

Retroelement expression levels were determined by applying a previously published customized pipeline [21]. First, repeat masked genomes were either downloaded directly from RepeatMasker.org (hg38 human genome assembly, RepeatMasker v 4.0.6, Dfam 2.0 and mm10 mouse genome assembly, RepeatMasker v 4.0.6 and Dfam 2.0) or generated (GRCz11 zebrafish genome assembly) using RepeatMasker v 4.1.0 and Dfam 3.5 [73]; http://www.repeatmasker.org). All repeat classes were annotated (simple repeats, low complexity sequences, satellites, transposons, retrotransposons, etc), and the resulting output files were converted to gene transfer file (GTF) format. This annotation file contains genomic coordinates, strand, conservation scores relative to consensus sequence, and relational information for each annotated repetitive element copy (repeat copy identifier generated by RepeatMasker, element name, repeat family, and repeat class). Please note that the nomenclature adopted for this paper is exemplified by the following: SINE (class), Alu (family), AluY (subfamily), AluYb8 (element), AluYb8 at chrX:115474404–115,474,722 (copy). Next, we identified and compiled the most highly conserved repeat element copies present in the genome (full length relative to the consensus for each element, fewer than 10 mismatches relative to consensus). Finally, we counted reads aligning to individual types of repeat elements (i.e. all copies of LINE1-Hs), reads aligning to repeat element families (i.e. all LINE1s) and reads aligning to repeat classes (i.e. LINEs). As the repetitive nature of these elements makes assigning RNA sequencing reads to individually identifiable genomic copies of these elements challenging or impossible, we only counted reads mapping entirely within a given repeat element type, family or class. Reads mapped ambiguously (multiply mapped read) to more than one copy of a specific type of repeat element were counted only once for data summarized at the level of repeat element. Similarly, a read mapping ambiguously to multiple types of repeat elements all within a given family or class of elements was counted only once for data summarized at the repeat family and class levels. Retroelement read count normalization was done using scale factors obtained from scPCG DESeq2 normalization.

### Differential gene expression analysis

scPCG and retroelement normalized read count tables from human cell lines with normalized read count < 20 across all replicates of both fractions were filtered out. The remaining read counts were used as input for differential gene expression analysis using edgeR v.3.34.0 as implemented in Galaxy ( [74], https://bioconductor.riken.jp/packages/3.0/bioc/html/edgeR.html) with default parameters (Benjamini and Hochberg *p*-value adjustment method; quasi-likelihood F-test). scPCGs and retroelements were considered significantly nuclear (log_2_FC > 0) or cytoplasmic (log_2_FC < 0) if FDR < 0.05.

### Metagene and heatmap analysis

Metagene and heatmap analyses were performed using the Deeptools 3.5.0 computeMatrix (computeMatrix scale-regions -b 1000 -a 1000 --binSize 10), plotProfile and plotHeatmap commands [75]. For human samples, metagene and heatmap analyses were generated for the subset of most highly conserved LINE1-Hs retroelement copies (see Methods: Retroelement expression analysis). For zebrafish samples, metagene and heatmap analyses were performed on the subset of full length retroelement copies with fewer than 10 mismatches or indels relative to a consensus sequence (see Methods: Retroelement expression analysis). These retroelement copies were further characterized based on intergenic or genic localization, and insertion orientation if nested within a protein coding gene. Only reads originating from the same strand as the retroelement copy are represented in these analyses. Either only uniquely assignable or both uniquely assignable and multiply mapped reads +/− 1 kb and across each analyzed individual repeat element copy are represented (see figure legends).

### Statistical analysis

Statistical analysis was performed using GraphPad Prism software. For analysis of retroelement and scPCG log_2_(Nuc/Cyt) ratios in RiboZero human brain tissue (Fig. 4), zebrafish embryo (Fig. 5A), polyA human brain tissue (Additional File 3A-B), mouse MIN6 cell line (Additional File 5B) and mouse liver tissue (Additional File 5B’), a one-tailed Mann-Whitney U (MWU) test was performed. *p*-values < 0.05 were considered significant. The median log_2_(Nuc/Cyt) ratios for retroelements and scPCGs and MWU *p*-values for all samples are listed in Additional File 9A-D.

## Supplementary Information

**Additional file 1.** Fractionation quality and DESeq2 normalization of RNA-sequencing data from PRJNA30709. (A) The graphs show the splicing indices of nuclear and cytoplasmic fractions for each cell line (A549, HUVEC, IMR90, K562, MCF7 and SK-N-SH) confirming good fractionation quality. The boundaries of the boxes denote 25th and 75th percentile and the whiskers mark the minimum and maximum values. The median is represented by the solid line inside the box. Note that the higher median splicing indices closer to 1 for cytoplasmic fractions indicate enrichment of fully spliced transcripts in the cytoplasm which in turn indicates good fractionation quality. In (B) A549, (C) HUVEC, (D) IMR90, (E) K562, (F) MCF7 and (G) SK-N-SH cell lines, all classes of retroelements are highly enriched in the nucleus (heatmaps on the left for each pair) compared to single-copy protein coding genes (scPCGs) (heatmaps on the right for each pair). Each row in the heatmaps represent a log_2_(Nuc/Cyt) expression value from a retroelement type or scPCG. Green indicates nuclear, red indicates cytoplasmic and black indicates no preferential localization. Retroelement type and scPCG expression values were normalized using DESeq2 (see Methods). Number of rows in each heatmap (n) is indicated. Note that the group of scPCGs and individual retroelement types analyzed for each cell line may be different (see Methods).

**Additional file 2.** Fractionation quality of RiboZero samples from PRJNA595606 and all samples from PRJNA434426. The graphs show the splicing indices of nuclear and cytoplasmic fractions for (A) Pre-natal prefrontal cortex, (B) adult dorsolateral prefrontal cortex, (C) fetal frontal cortex, (D) fetal cerebellum and (E) adult frontal cortex samples confirming good fractionation quality. The boundaries of the boxes denote 25th and 75th percentile and the whiskers mark the minimum and maximum values. The median is represented by the solid line inside the box.

**Additional file 3 **Comparison of retroelement type and scPCG log_2_(Nuc/Cyt) ratios and fractionation quality of polyA samples from PRJNA595606. RNA-sequencing data from libraries prepared by polyA selection (polyA) from nuclear (Nuc) and cytoplasmic (Cyt) fractions of human brain tissue was analyzed to study retroelement RNA localization in vivo*.* Retroelement RNAs (Retro) are significantly nuclear localized (MWU, *** = *p* < 0.001, **** = *p* < 0.0001) unlike single-copy protein coding genes (scPCGs) in (A) Pre-natal prefrontal cortex (Br5339, Br5340, Br5341) and (B) Adult dorsolateral prefrontal cortex (Br2046, Br2074). The graphs in (A and B) plot log_2_(Nuc/Cyt) ratios for retroelement RNAs, calculated by type (see Methods) and scPCGs for each brain sample. The boundaries of the boxes denote 25th and 75th percentile and the whiskers mark the minimum and maximum values. The median log_2_(Nuc/Cyt) ratios are represented by the solid line inside the box. RNAs with log_2_(Nuc/Cyt) values greater than and less than 0 are considered nuclear or cytoplasmic respectively. Splicing indices of nuclear and cytoplasmic fractions for (C) Pre-natal prefrontal cortex and (D) Adult dorsolateral prefrontal cortex tissue samples are shown. The boundaries of the boxes denote 25th and 75th percentile and the whiskers mark the minimum and maximum values. The median is represented by the solid line inside the box.

**Additional file 4.** Fractionation quality of samples from PRJNA599208 and distribution of retroelements across developmental stages. (A) Splicing indices of nuclear and cytoplasmic fractions for 64 cell, 256 cell, 1000 cell, Dome and Shield stages of zebrafish embryos confirming good fractionation quality. The boundaries of the boxes denote 25th and 75th percentile and the whiskers mark the minimum and maximum values. The median is represented by the solid line inside the box. Note that median splicing indices are similar for nuclear and cytoplasmic fractions when the transcripts are maternally sourced. However, during MZT (1000 cell stage) and ZGA (Dome and Shield stages) median cytoplasmic splicing indices are higher. (B) The percentage of each class of expressed retroelements (LINE, LTR, Retroposon, SINE) does not change considerably during zebrafish embryo development.

**Additional file 5 **Mouse MIN6 and liver tissue samples also show nuclear enrichment of retroelement RNAs despite high levels of 5p degradation of nuclear fractions. RNA-sequencing data from nuclear (Nuc) and cytoplasmic (Cyt) fractions of mouse cell line MIN6 and mouse liver tissue (PRJNA298634) was analyzed to study localization of retroelement RNAs. Nuclear fractions of both samples in this dataset did not meet our quality metrics and showed pronounced 5p degradation. Nevertheless, these samples were analyzed using our pipeline to determine retroelement RNA localization. For (A) MIN6 and (A’) Liver tissue, normalized read coverage of the 500 most highly expressed genes in each replicate is plotted against normalized transcript position from 5p to 3p. For both MIN6 and liver samples, the nuclear replicates show a 3p skew indicating degradation at the 5p end of transcripts. In (B) MIN6 and (B′) Liver tissue, retroelement RNAs are significantly nuclear localized (MWU, *p* < 0.0001) compared to single-copy protein coding genes (scPCGs). The graphs show log_2_(Nuc/Cyt) ratios plotted for types of retroelements and scPCGs for each sample. The boundaries of the boxes denote 25th and 75th percentile and the whiskers mark the minimum and maximum values. The median log_2_(Nuc/Cyt) ratios are represented by the solid line inside the box. Transcripts with log_2_(Nuc/Cyt) values greater than 0 are considered nuclear and those less than 0 are considered cytoplasmic. Splicing indices of nuclear and cytoplasmic fractions from (C) MIN6 and (C′) Liver tissue indicates fractionation quality. The boundaries of the boxes denote 25th and 75th percentile and the whiskers mark the minimum and maximum values. The median is represented by the solid line inside the box. Note that the median splicing indices are indicative of good quality of fractionation.

**Additional file 6.** Primers used in this study. Names and sequences of primers targeting 5pUTR and 3pUTR of L1, Malat1 and β-actin.

**Additional file 7.** Quality Control metrics (including input reads, unique reads, PCR duplicates) of all datasets analyzed in this study.

**Additional file 8.** RNA metrics. Normalized read coverage at normalized transcript position for the 500 most highly expressed genes of all datasets analyzed in this study.

**Additional file 9 **Mann-Whitney U (MWU) results. MWU *p*-values and number of retroelements and scPCGs analyzed.

**Additional file 10.** Relative abundance of L1-Hs expression in nuclear and cytoplasmic fractions. The graphs in A-E’ show normalized expression (log_10_ transformed) of scPCGs in Nuc and Cyt fractions from A) Fetal frontal cortex, B) Fetal cerebellum, C) Adult frontal cortex, D-D′) Pre-natal prefrontal cortex and E-E’) Adult dorsolateral prefrontal cortex. The red dots in each sample represents L1-Hs expression (normalized, log_10_ transformed). The boundaries of the boxes denote 25th and 75th percentile and the whiskers mark the minimum and maximum expression values. The median expression is represented by the solid line inside the box. Note that Rz (RiboZero) and pA (polyA) refer to sample library preparation method.

**Additional file 11.** Metagene and heatmap analyses of intergenic, genic antisense and genic sense full length L1-Hs genomic copies using unique and multi-mapped reads from human adult dorsolateral prefrontal cortex and pre-natal prefrontal cortex samples (PRJNA595606) prepared using PolyA enrichment. Heatmaps of unique and multiply mapped reads from intergenic (second panel), genic antisense (third panel) and genic sense (bottom panel) full length L1-Hs retroelement copies +/− 1 kb. Sample labels and nuclear or cytoplasmic fraction are indicated above each heatmap. Heatmap color scales represent the TMM normalized signal from RNA sequencing reads originating from the same strand as genomic negative strand (A) or genomic positive strand (B) full length L1-Hs copies. Red asterisks indicate individual L1-Hs copies used in readthrough transcription RT-PCR assays (see Fig. 1D). Please see Additional File 15 for genomic coordinates of L1-Hs copies included in these heatmaps. Metagene summary analyses (average signal by relative position) for each sample and grouping are also presented (top panel, dark blue = average intergenic L1-Hs signal, light blue = average nested antisense L1-Hs signal, yellow = average nested sense L1-Hs signal).

**Additional file 12.** Metagene and heatmap analysis of intergenic, genic antisense and genic sense full length L1-Hs copies displaying only uniquely assignable reads from human adult dorsolateral prefrontal cortex and pre-natal prefrontal cortex samples (PRJNA595606) prepared using PolyA library prep. Heatmaps of unique reads from intergenic (second panel), genic antisense (third panel) and genic sense (bottom panel) full length L1-Hs copies +/− 1 kb. Sample labels and nuclear or cytoplasmic fraction are indicated above each heatmap. Heatmap color scales represent the TMM normalized signal from RNA sequencing reads originating from the same strand as genomic negative strand (A) or genomic positive strand (B) full length L1-Hs copies. Red asterisks indicate individual L1-Hs copies used in readthrough transcription RT-PCR assays (see Fig. 1D). Please see Additional File 15 for genomic coordinates of L1-Hs copies included in these heatmaps. Metagene summary analyses (average signal by relative position) for each sample and grouping are also presented (top panel, dark blue = average intergenic L1-Hs signal, light blue = average nested antisense L1-Hs signal, yellow = average nested sense L1-Hs signal).

**Additional file 13.** Metagene and heatmap analysis of intergenic, genic antisense and genic sense full length L1-Hs copies using unique and multi-mapped reads from human adult dorsolateral prefrontal cortex and pre-natal prefrontal cortex samples (PRJNA595606) prepared using ribosomal RNA depletion. Heatmaps of unique and multiply mapped reads from intergenic (second panel), genic antisense (third panel) and genic sense (bottom panel) full length L1-Hs copies +/− 1 kb. Sample labels and nuclear or cytoplasmic fraction are indicated above each heatmap. Heatmap color scales represent the TMM normalized signal from RNA sequencing reads originating from the same strand as genomic negative strand (A) or genomic positive strand (B) full length L1-Hs copies. Red asterisks indicate individual L1-Hs copies used in readthrough transcription RT-PCR assays (see Fig. 1D). Please see Additional File 15 for genomic coordinates of L1-Hs copies included in these heatmaps. Note that heatmap color scales were selected to best display the range of presented data, therefore, color scales for heatmaps containing only unique reads (Additional File 14, scale range 0–5 normalized reads) differ from the color scales for all mappable reads (Additional File 13, scale range 0–60 normalized reads). Metagene summary analyses (average normalized signal by relative position) for each sample and grouping are also presented (top panel, dark blue = average intergenic L1-Hs signal, light blue = average nested antisense L1-Hs signal, yellow = average nested sense L1-Hs signal).

**Additional file 14.** Metagene and heatmap analysis of intergenic, genic antisense and genic sense full length L1-Hs copies displaying only uniquely assignable reads from human adult dorsolateral prefrontal cortex and pre-natal prefrontal cortex samples (PRJNA595606) prepared using ribosomal RNA depletion. Heatmaps of unique reads from intergenic (second panel), genic antisense (third panel) and genic sense (bottom panel) full length L1-Hs copies +/− 1 kb. Sample labels and nuclear or cytoplasmic fraction are indicated above each heatmap. Heatmap color scales represent the normalized signal from RNA sequencing reads originating from the same strand as genomic negative strand (A) or genomic positive strand (B) full length L1-Hs copies. Red asterisks indicate individual L1-Hs copies used in readthrough transcription RT-PCR assays (see Fig. 1D). Please see Additional File 15 for genomic coordinates of L1-Hs copies included in these heatmaps. Note that heatmap color scales were selected to best display the range of presented data, therefore, color scales for heatmaps containing only unique reads (Additional File 14, scale range 0–5 normalized reads) differ from the color scales for all mappable reads (Additional File 13, scale range 0–60 normalized reads). Metagene summary analyses (average normalized signal by relative position) for each sample and grouping are also presented (top panel, dark blue = average intergenic L1-Hs signal, light blue = average nested antisense L1-Hs signal, yellow = average nested sense L1-Hs signal).

**Additional file 15.** Genomic coordinates of L1-Hs copies included in heatmap analyses. Genomic coordinates of L1-Hs copies corresponding to hg38 human genome assembly included in heatmap analyses grouped as intergenic, genic sense and genic antisense for both genomic negative and positive strands. The three L1-Hs copies tested in the readthrough transcription assays in Fig. 1D are highlighted and annotated.

**Additional file 16.** Metagene and heatmap analysis of intergenic, genic antisense and genic sense zebrafish retroelements from five different embryonic developmental stages (PRJNA599208) using unique and multi-mapped reads prepared using ribosomal RNA depletion protocols. Heatmaps of unique and multiply mapped reads from intergenic (second panel), genic antisense (third panel) and genic sense (bottom panel) zebrafish retroelement copies. Only the subset of full length retroelement copies with fewer than 10 mismatches or indels relative to their consensus sequence are included (see Additional File 17). Sample labels are indicated above each heatmap (from left to right- 64c_Cyto, 64c_Nuc, 256c_Cyto, 256c_Nuc, 1000c_Cyto, 1000c_Nuc, Dome_Cyto, Dome-Nuc, Shield_Cyto, Shield_Nuc where c denotes cells, Cyto denotes cytoplasmic and Nuc denotes nuclear fraction). Heatmap color scales represent the TMM normalized signal from RNA sequencing reads originating from the same strand as genomic negative strand (A) or genomic positive strand (B) retroelement copies. Metagene summary analyses (average normalized signal by relative position) for each sample and grouping are also presented (top panel, dark blue = average intergenic retroelement signal, light blue = average nested antisense retroelement signal, yellow = average nested sense retroelement signal). Please see Additional File 17 for genomic coordinates of retroelements included in these heatmaps.

**Additional file 17.** Genomic coordinates of zebrafish retroelement copies included in heatmap analyses. Genomic coordinates of zebrafish retroelements corresponding to zf11 zebrafish genome assembly included in heatmap analyses grouped as intergenic, genic sense and genic antisense for both genomic negative and positive strands.

## Data Availability

The datasets analyzed in the current study are available in the NCBI Sequence Read Archive repository, [PRJNA30709], [PRJNA595606], [PRJNA434426], [PRJNA599208] and [PRJNA298634].
