# Supplementary figures and images for: Generalized nuclear localization of retroelement transcripts
Source: Mob DNA. 2022 Dec 2;13:30. doi: 10.1186/s13100-022-00287-x (PMC9717504; doi:10.1186/s13100-022-00287-x)

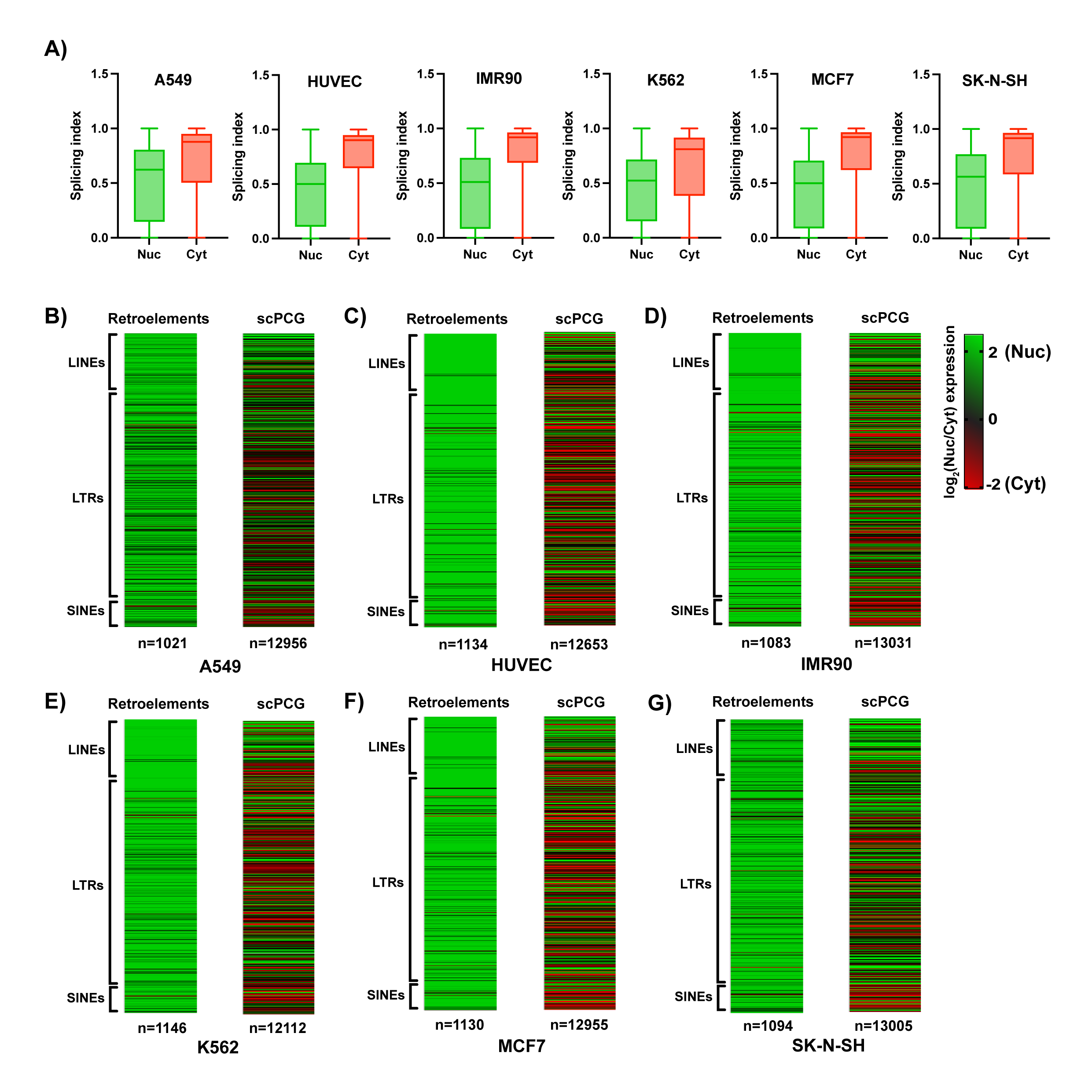

Supplement: Supplementary file 1 — Additional file 1. Fractionation quality and DESeq2 normalization of RNA-sequencing data from PRJNA30709. (A) The graphs show the splicing indices of nuclear and cytoplasmic fractions for each cell line (A549, HUVEC, IMR90, K562, MCF7 and SK-N-SH) confirming good fractionation quality. The boundaries of the boxes denote 25th and 75th percentile and the whiskers mark the minimum and maximum values. The median is represented by the solid line inside the box. Note that the higher median splicing indices closer to 1 for cytoplasmic fractions indicate enrichment of fully spliced transcripts in the cytoplasm which in turn indicates good fractionation quality. In (B) A549, (C) HUVEC, (D) IMR90, (E) K562, (F) MCF7 and (G) SK-N-SH cell lines, all classes of retroelements are highly enriched in the nucleus (heatmaps on the left for each pair) compared to single-copy protein coding genes (scPCGs) (heatmaps on the right for each pair). Each row in the heatmaps represent a log2(Nuc/Cyt) expression value from a retroelement type or scPCG. Green indicates nuclear, red indicates cytoplasmic and black indicates no preferential localization. Retroelement type and scPCG expression values were normalized using DESeq2 (see Methods). Number of rows in each heatmap (n) is indicated. Note that the group of scPCGs and individual retroelement types analyzed for each cell line may be different (see Methods). [file 13100_2022_287_MOESM1_ESM.tif]

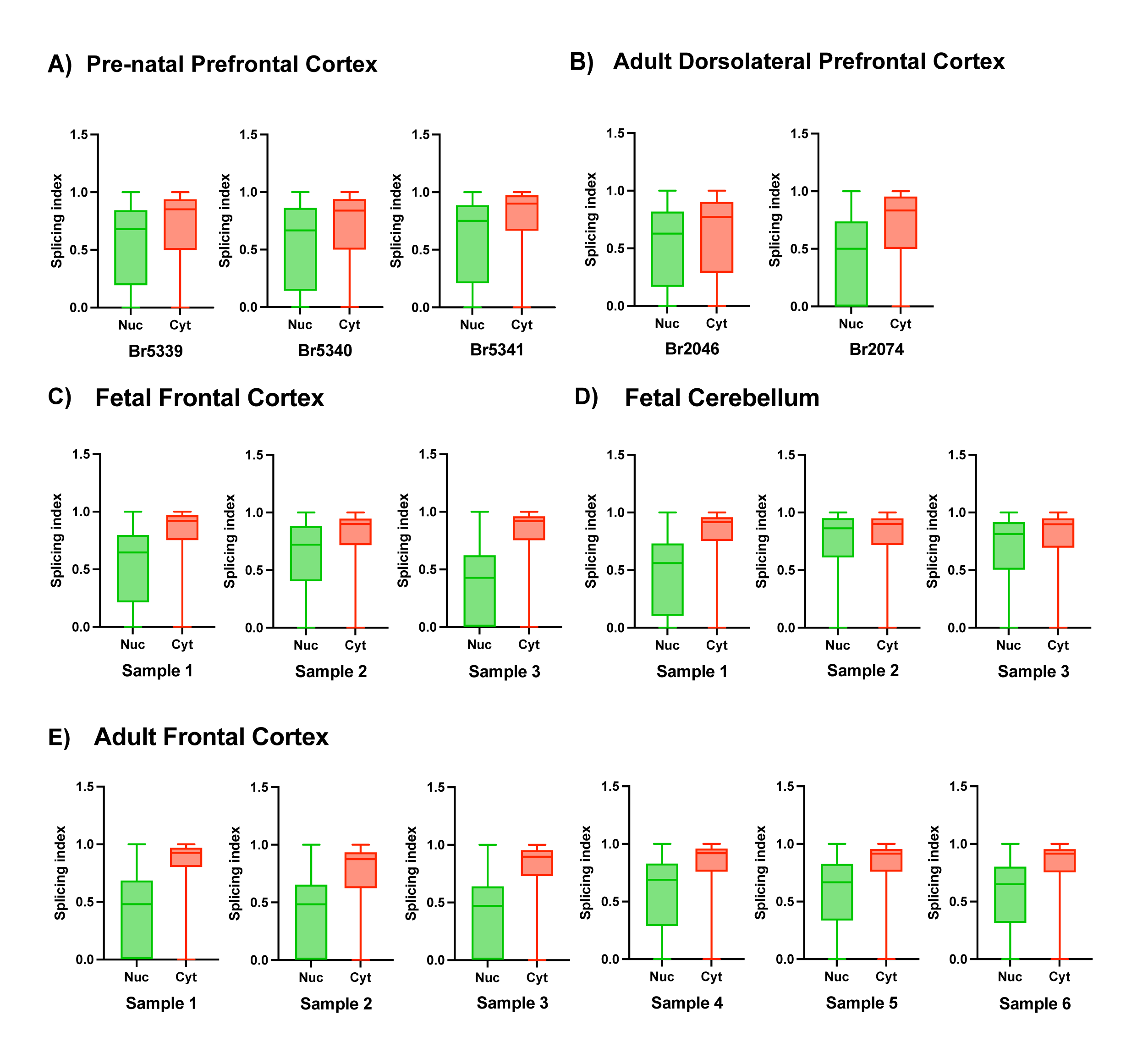

Supplement: Supplementary file 2 — Additional file 2. Fractionation quality of RiboZero samples from PRJNA595606 and all samples from PRJNA434426. The graphs show the splicing indices of nuclear and cytoplasmic fractions for (A) Pre-natal prefrontal cortex, (B) adult dorsolateral prefrontal cortex, (C) fetal frontal cortex, (D) fetal cerebellum and (E) adult frontal cortex samples confirming good fractionation quality. The boundaries of the boxes denote 25th and 75th percentile and the whiskers mark the minimum and maximum values. The median is represented by the solid line inside the box. [file 13100_2022_287_MOESM2_ESM.tif]

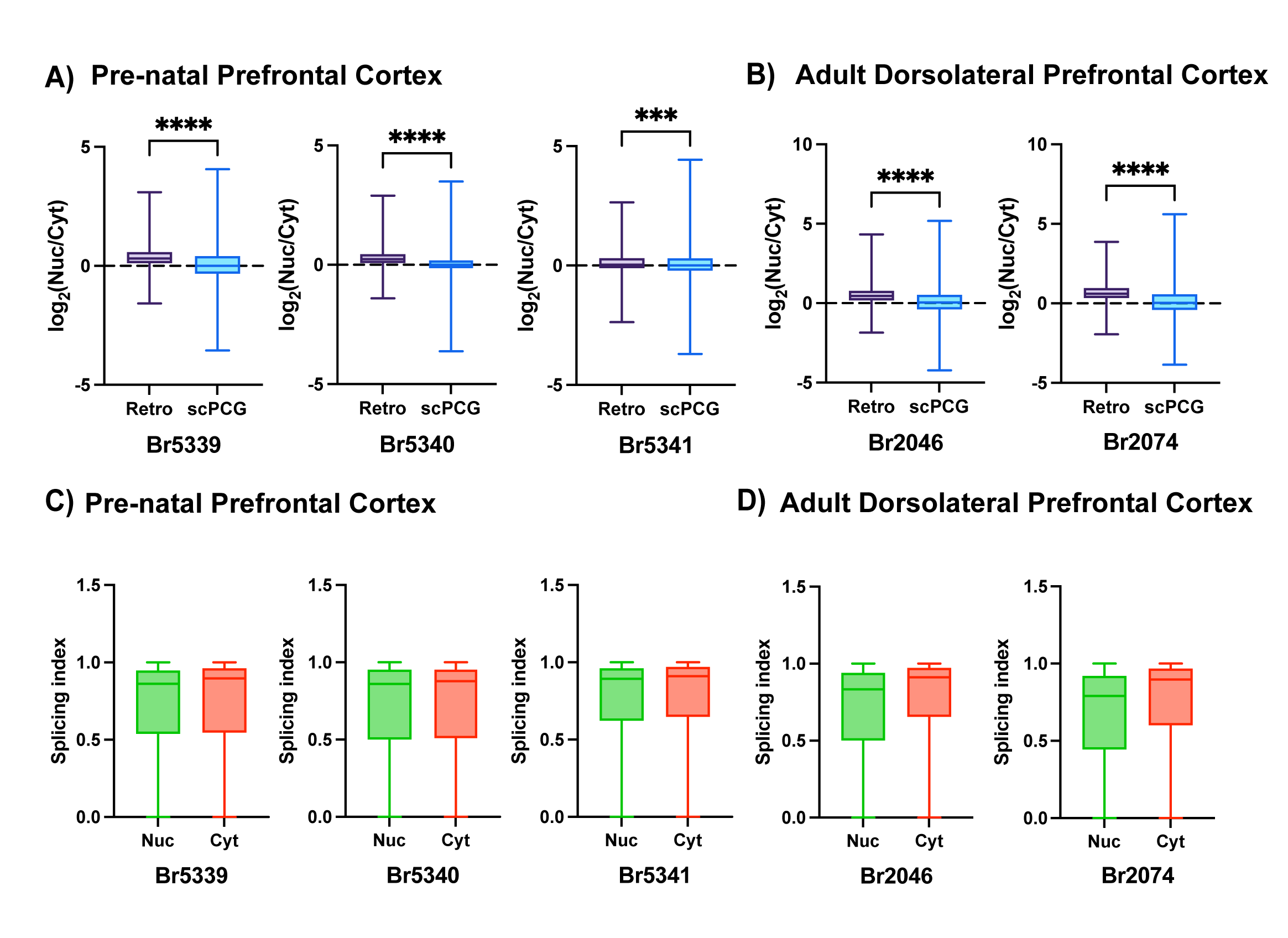

Supplement: Supplementary file 3 — Additional file 3 Comparison of retroelement type and scPCG log2(Nuc/Cyt) ratios and fractionation quality of polyA samples from PRJNA595606. RNA-sequencing data from libraries prepared by polyA selection (polyA) from nuclear (Nuc) and cytoplasmic (Cyt) fractions of human brain tissue was analyzed to study retroelement RNA localization in vivo. Retroelement RNAs (Retro) are significantly nuclear localized (MWU, *** = p < 0.001, **** = p < 0.0001) unlike single-copy protein coding genes (scPCGs) in (A) Pre-natal prefrontal cortex (Br5339, Br5340, Br5341) and (B) Adult dorsolateral prefrontal cortex (Br2046, Br2074). The graphs in (A and B) plot log2(Nuc/Cyt) ratios for retroelement RNAs, calculated by type (see Methods) and scPCGs for each brain sample. The boundaries of the boxes denote 25th and 75th percentile and the whiskers mark the minimum and maximum values. The median log2(Nuc/Cyt) ratios are represented by the solid line inside the box. RNAs with log2(Nuc/Cyt) values greater than and less than 0 are considered nuclear or cytoplasmic respectively. Splicing indices of nuclear and cytoplasmic fractions for (C) Pre-natal prefrontal cortex and (D) Adult dorsolateral prefrontal cortex tissue samples are shown. The boundaries of the boxes denote 25th and 75th percentile and the whiskers mark the minimum and maximum values. The median is represented by the solid line inside the box. [file 13100_2022_287_MOESM3_ESM.tif]

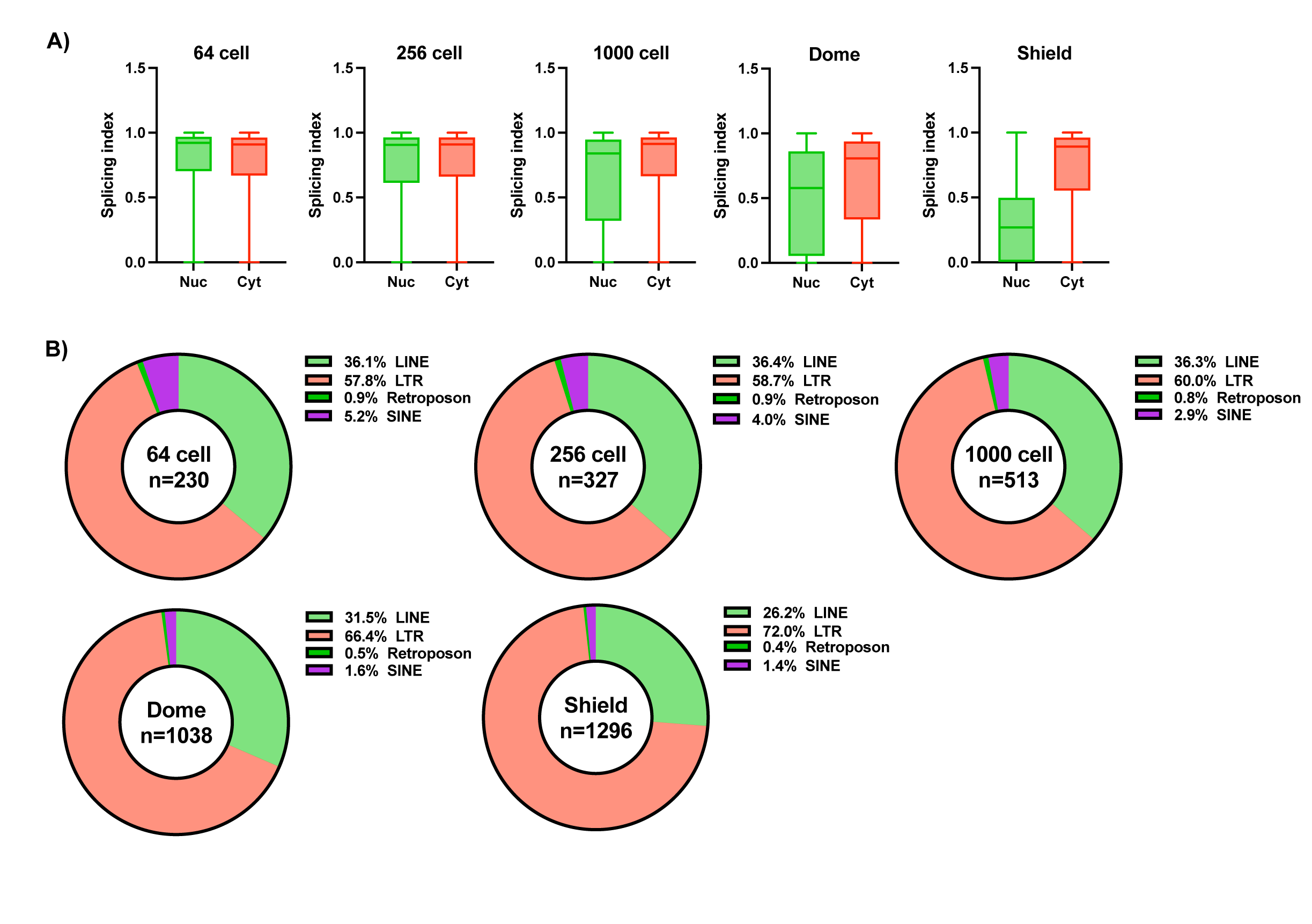

Supplement: Supplementary file 4 — Additional file 4. Fractionation quality of samples from PRJNA599208 and distribution of retroelements across developmental stages. (A) Splicing indices of nuclear and cytoplasmic fractions for 64 cell, 256 cell, 1000 cell, Dome and Shield stages of zebrafish embryos confirming good fractionation quality. The boundaries of the boxes denote 25th and 75th percentile and the whiskers mark the minimum and maximum values. The median is represented by the solid line inside the box. Note that median splicing indices are similar for nuclear and cytoplasmic fractions when the transcripts are maternally sourced. However, during MZT (1000 cell stage) and ZGA (Dome and Shield stages) median cytoplasmic splicing indices are higher. (B) The percentage of each class of expressed retroelements (LINE, LTR, Retroposon, SINE) does not change considerably during zebrafish embryo development. [file 13100_2022_287_MOESM4_ESM.tif]

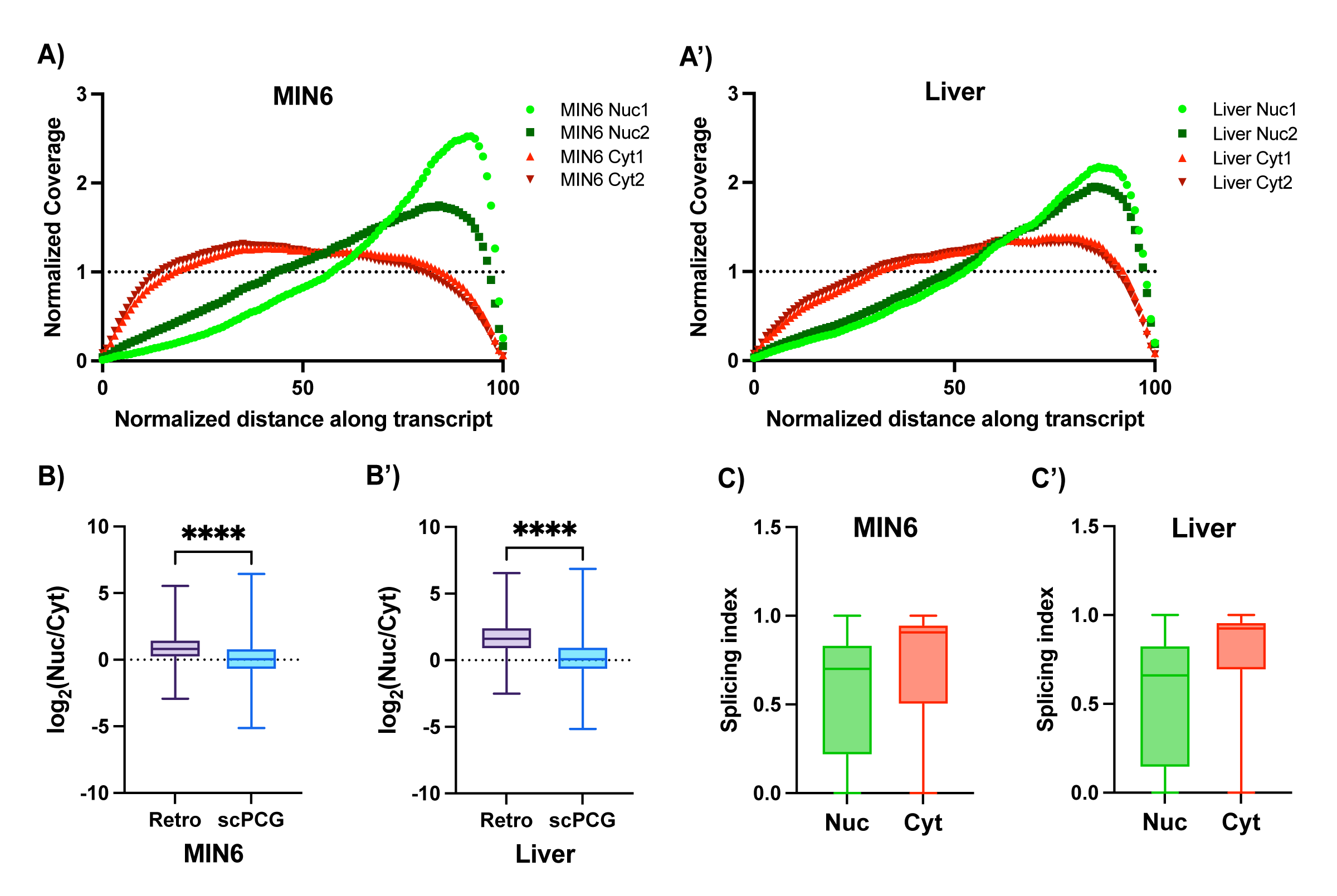

Supplement: Supplementary file 5 — Additional file 5 Mouse MIN6 and liver tissue samples also show nuclear enrichment of retroelement RNAs despite high levels of 5p degradation of nuclear fractions. RNA-sequencing data from nuclear (Nuc) and cytoplasmic (Cyt) fractions of mouse cell line MIN6 and mouse liver tissue (PRJNA298634) was analyzed to study localization of retroelement RNAs. Nuclear fractions of both samples in this dataset did not meet our quality metrics and showed pronounced 5p degradation. Nevertheless, these samples were analyzed using our pipeline to determine retroelement RNA localization. For (A) MIN6 and (A’) Liver tissue, normalized read coverage of the 500 most highly expressed genes in each replicate is plotted against normalized transcript position from 5p to 3p. For both MIN6 and liver samples, the nuclear replicates show a 3p skew indicating degradation at the 5p end of transcripts. In (B) MIN6 and (B′) Liver tissue, retroelement RNAs are significantly nuclear localized (MWU, p < 0.0001) compared to single-copy protein coding genes (scPCGs). The graphs show log2(Nuc/Cyt) ratios plotted for types of retroelements and scPCGs for each sample. The boundaries of the boxes denote 25th and 75th percentile and the whiskers mark the minimum and maximum values. The median log2(Nuc/Cyt) ratios are represented by the solid line inside the box. Transcripts with log2(Nuc/Cyt) values greater than 0 are considered nuclear and those less than 0 are considered cytoplasmic. Splicing indices of nuclear and cytoplasmic fractions from (C) MIN6 and (C′) Liver tissue indicates fractionation quality. The boundaries of the boxes denote 25th and 75th percentile and the whiskers mark the minimum and maximum values. The median is represented by the solid line inside the box. Note that the median splicing indices are indicative of good quality of fractionation. [file 13100_2022_287_MOESM5_ESM.tif]

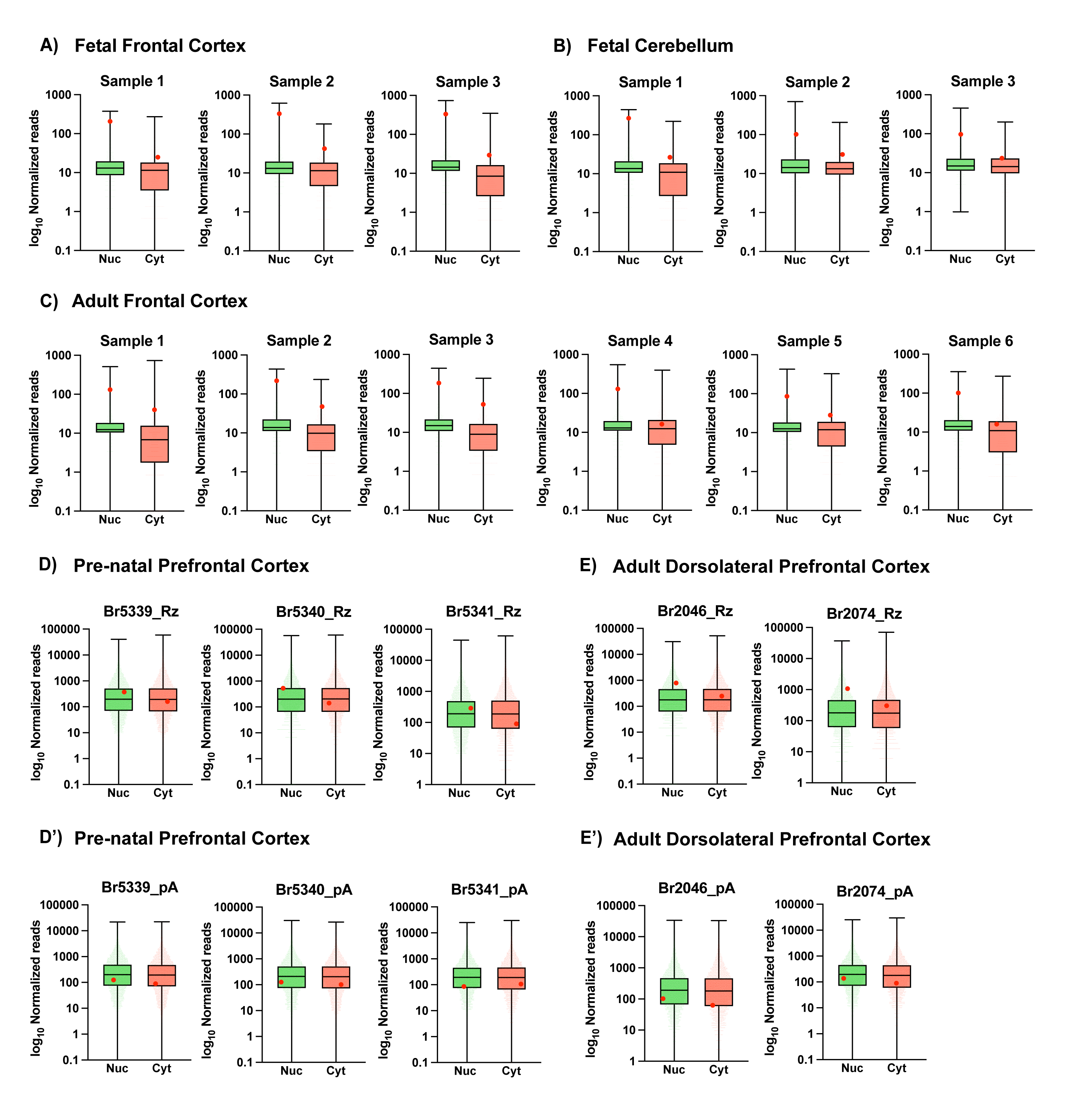

Supplement: Supplementary file 10 — Additional file 10. Relative abundance of L1-Hs expression in nuclear and cytoplasmic fractions. The graphs in A-E’ show normalized expression (log10 transformed) of scPCGs in Nuc and Cyt fractions from A) Fetal frontal cortex, B) Fetal cerebellum, C) Adult frontal cortex, D-D′) Pre-natal prefrontal cortex and E-E’) Adult dorsolateral prefrontal cortex. The red dots in each sample represents L1-Hs expression (normalized, log10 transformed). The boundaries of the boxes denote 25th and 75th percentile and the whiskers mark the minimum and maximum expression values. The median expression is represented by the solid line inside the box. Note that Rz (RiboZero) and pA (polyA) refer to sample library preparation method. [file 13100_2022_287_MOESM10_ESM.tif]

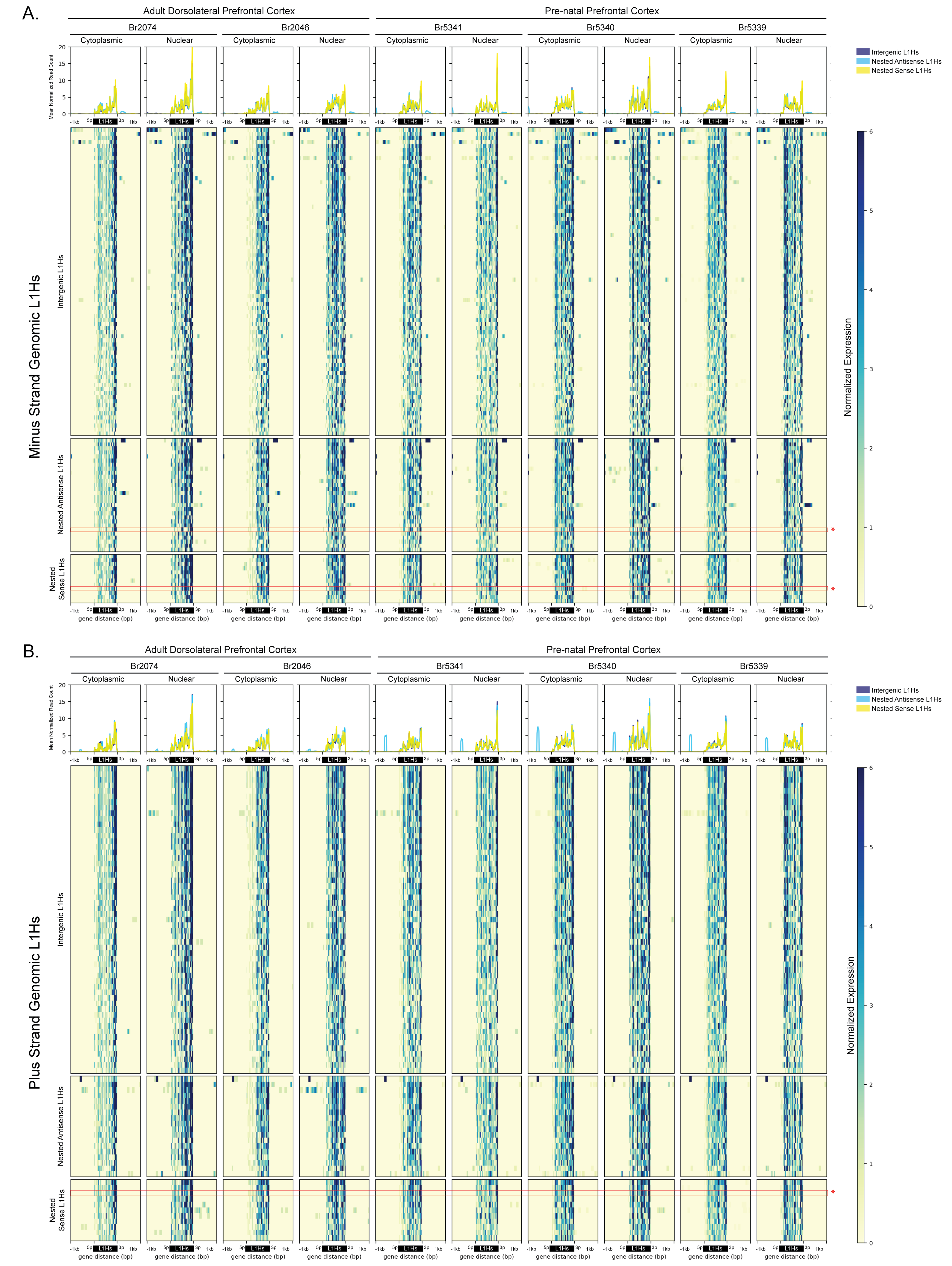

Supplement: Supplementary file 11 — Additional file 11. Metagene and heatmap analyses of intergenic, genic antisense and genic sense full length L1-Hs genomic copies using unique and multi-mapped reads from human adult dorsolateral prefrontal cortex and pre-natal prefrontal cortex samples (PRJNA595606) prepared using PolyA enrichment. Heatmaps of unique and multiply mapped reads from intergenic (second panel), genic antisense (third panel) and genic sense (bottom panel) full length L1-Hs retroelement copies +/− 1 kb. Sample labels and nuclear or cytoplasmic fraction are indicated above each heatmap. Heatmap color scales represent the TMM normalized signal from RNA sequencing reads originating from the same strand as genomic negative strand (A) or genomic positive strand (B) full length L1-Hs copies. Red asterisks indicate individual L1-Hs copies used in readthrough transcription RT-PCR assays (see Fig. 1D). Please see Additional File 15 for genomic coordinates of L1-Hs copies included in these heatmaps. Metagene summary analyses (average signal by relative position) for each sample and grouping are also presented (top panel, dark blue = average intergenic L1-Hs signal, light blue = average nested antisense L1-Hs signal, yellow = average nested sense L1-Hs signal). [file 13100_2022_287_MOESM11_ESM.tif]

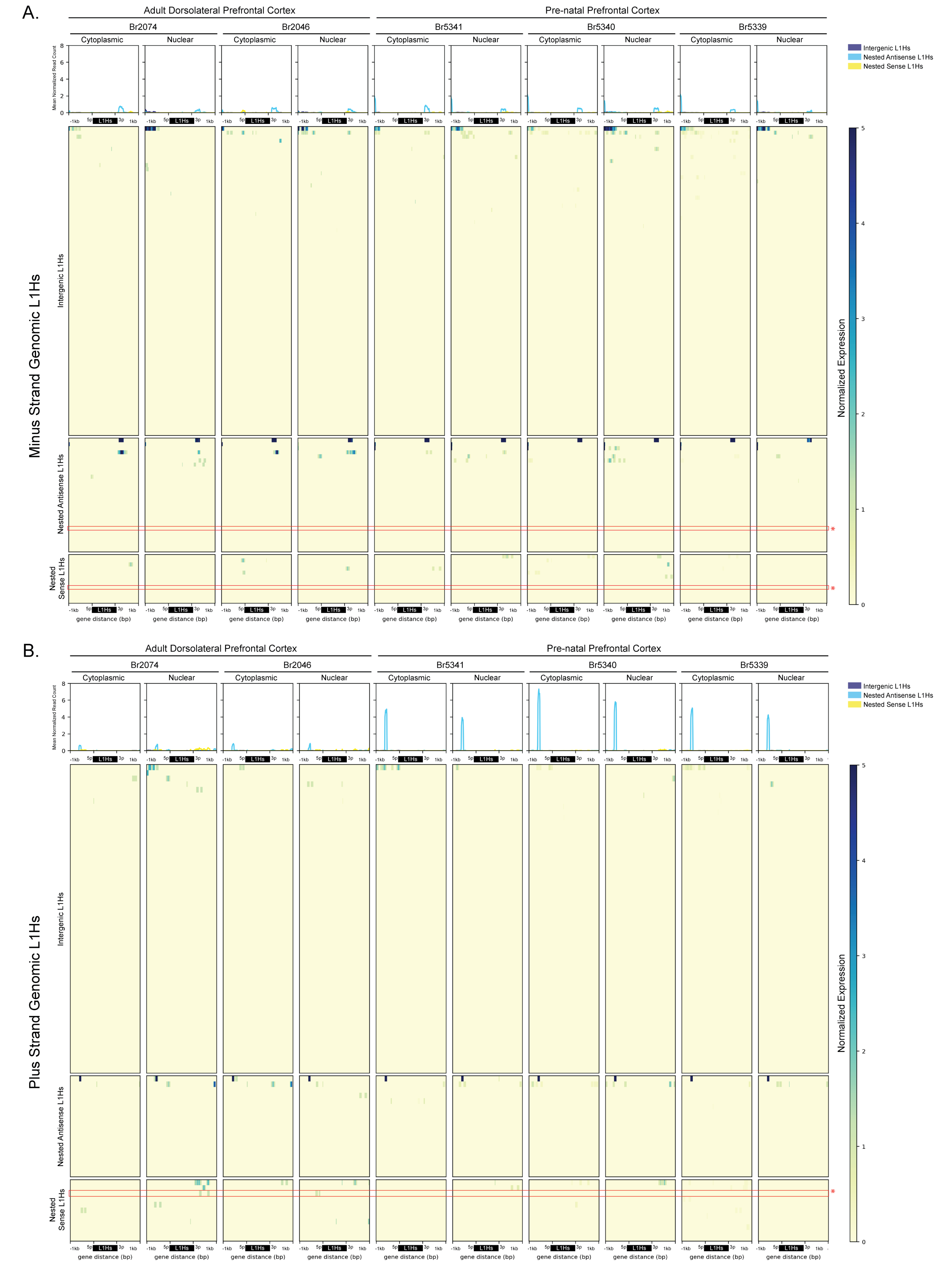

Supplement: Supplementary file 12 — Additional file 12. Metagene and heatmap analysis of intergenic, genic antisense and genic sense full length L1-Hs copies displaying only uniquely assignable reads from human adult dorsolateral prefrontal cortex and pre-natal prefrontal cortex samples (PRJNA595606) prepared using PolyA library prep. Heatmaps of unique reads from intergenic (second panel), genic antisense (third panel) and genic sense (bottom panel) full length L1-Hs copies +/− 1 kb. Sample labels and nuclear or cytoplasmic fraction are indicated above each heatmap. Heatmap color scales represent the TMM normalized signal from RNA sequencing reads originating from the same strand as genomic negative strand (A) or genomic positive strand (B) full length L1-Hs copies. Red asterisks indicate individual L1-Hs copies used in readthrough transcription RT-PCR assays (see Fig. 1D). Please see Additional File 15 for genomic coordinates of L1-Hs copies included in these heatmaps. Metagene summary analyses (average signal by relative position) for each sample and grouping are also presented (top panel, dark blue = average intergenic L1-Hs signal, light blue = average nested antisense L1-Hs signal, yellow = average nested sense L1-Hs signal). [file 13100_2022_287_MOESM12_ESM.tif]

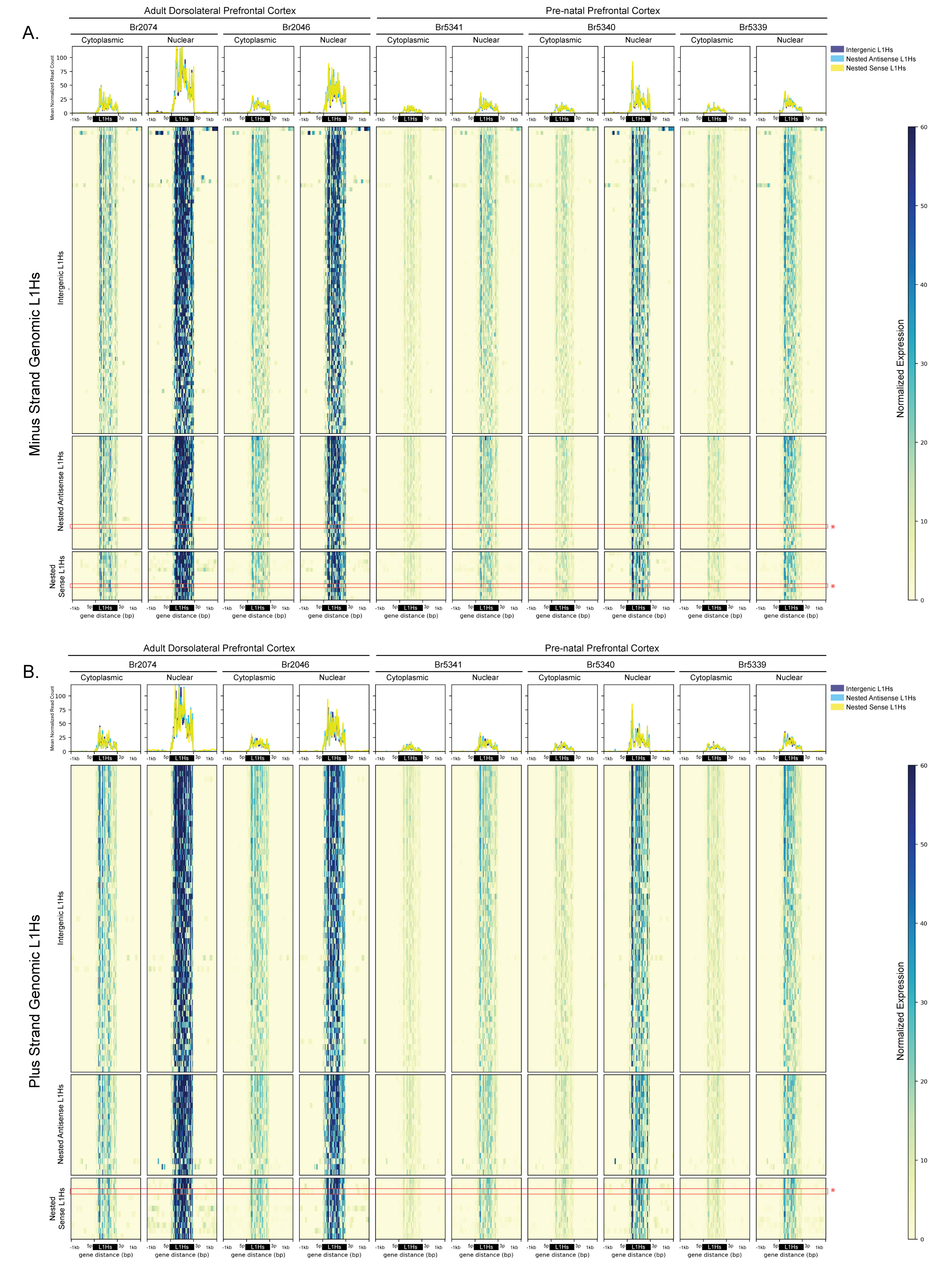

Supplement: Supplementary file 13 — Additional file 13. Metagene and heatmap analysis of intergenic, genic antisense and genic sense full length L1-Hs copies using unique and multi-mapped reads from human adult dorsolateral prefrontal cortex and pre-natal prefrontal cortex samples (PRJNA595606) prepared using ribosomal RNA depletion. Heatmaps of unique and multiply mapped reads from intergenic (second panel), genic antisense (third panel) and genic sense (bottom panel) full length L1-Hs copies +/− 1 kb. Sample labels and nuclear or cytoplasmic fraction are indicated above each heatmap. Heatmap color scales represent the TMM normalized signal from RNA sequencing reads originating from the same strand as genomic negative strand (A) or genomic positive strand (B) full length L1-Hs copies. Red asterisks indicate individual L1-Hs copies used in readthrough transcription RT-PCR assays (see Fig. 1D). Please see Additional File 15 for genomic coordinates of L1-Hs copies included in these heatmaps. Note that heatmap color scales were selected to best display the range of presented data, therefore, color scales for heatmaps containing only unique reads (Additional File 14, scale range 0–5 normalized reads) differ from the color scales for all mappable reads (Additional File 13, scale range 0–60 normalized reads). Metagene summary analyses (average normalized signal by relative position) for each sample and grouping are also presented (top panel, dark blue = average intergenic L1-Hs signal, light blue = average nested antisense L1-Hs signal, yellow = average nested sense L1-Hs signal). [file 13100_2022_287_MOESM13_ESM.tif]

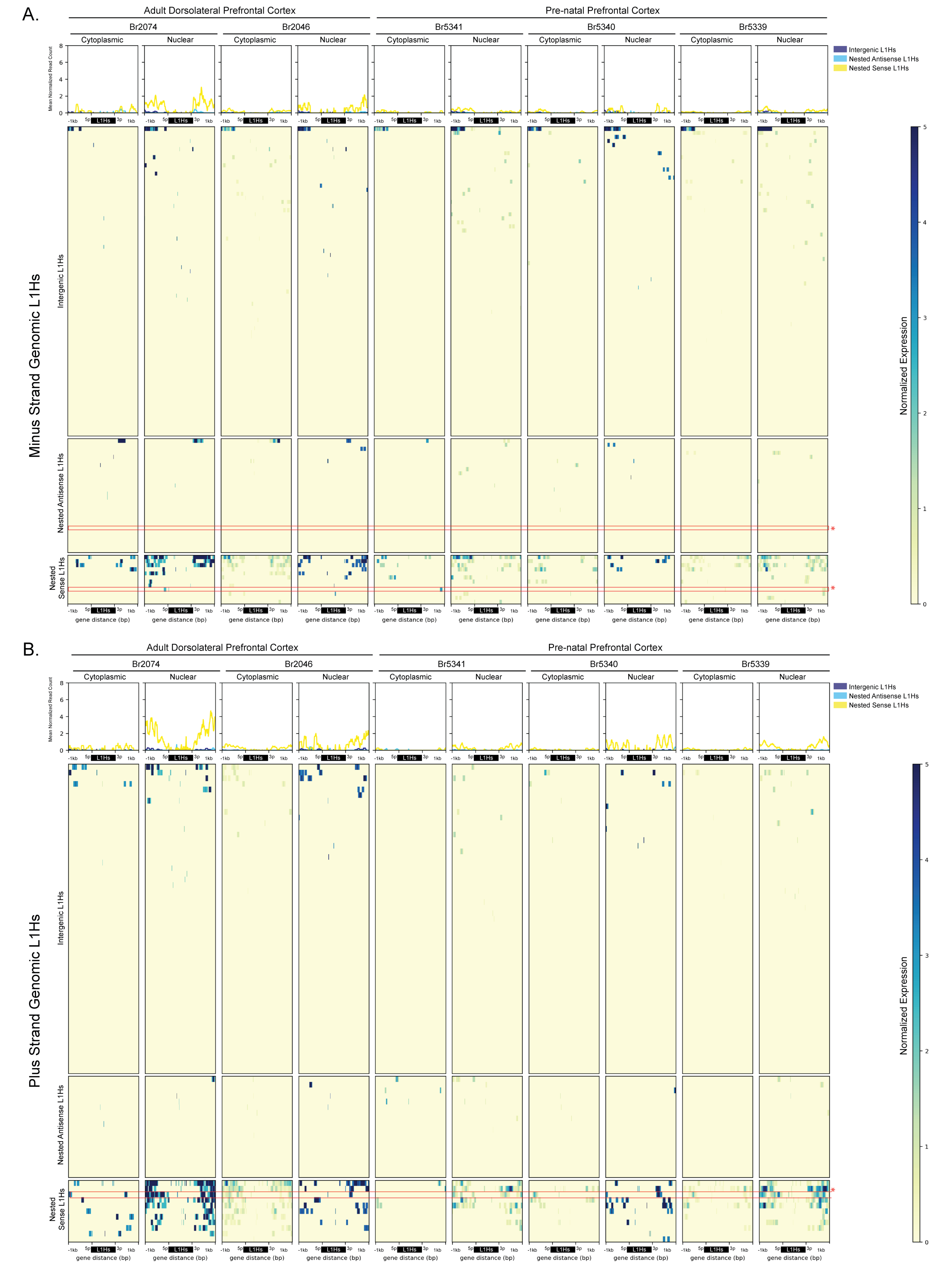

Supplement: Supplementary file 14 — Additional file 14. Metagene and heatmap analysis of intergenic, genic antisense and genic sense full length L1-Hs copies displaying only uniquely assignable reads from human adult dorsolateral prefrontal cortex and pre-natal prefrontal cortex samples (PRJNA595606) prepared using ribosomal RNA depletion. Heatmaps of unique reads from intergenic (second panel), genic antisense (third panel) and genic sense (bottom panel) full length L1-Hs copies +/− 1 kb. Sample labels and nuclear or cytoplasmic fraction are indicated above each heatmap. Heatmap color scales represent the normalized signal from RNA sequencing reads originating from the same strand as genomic negative strand (A) or genomic positive strand (B) full length L1-Hs copies. Red asterisks indicate individual L1-Hs copies used in readthrough transcription RT-PCR assays (see Fig. 1D). Please see Additional File 15 for genomic coordinates of L1-Hs copies included in these heatmaps. Note that heatmap color scales were selected to best display the range of presented data, therefore, color scales for heatmaps containing only unique reads (Additional File 14, scale range 0–5 normalized reads) differ from the color scales for all mappable reads (Additional File 13, scale range 0–60 normalized reads). Metagene summary analyses (average normalized signal by relative position) for each sample and grouping are also presented (top panel, dark blue = average intergenic L1-Hs signal, light blue = average nested antisense L1-Hs signal, yellow = average nested sense L1-Hs signal). [file 13100_2022_287_MOESM14_ESM.tif]

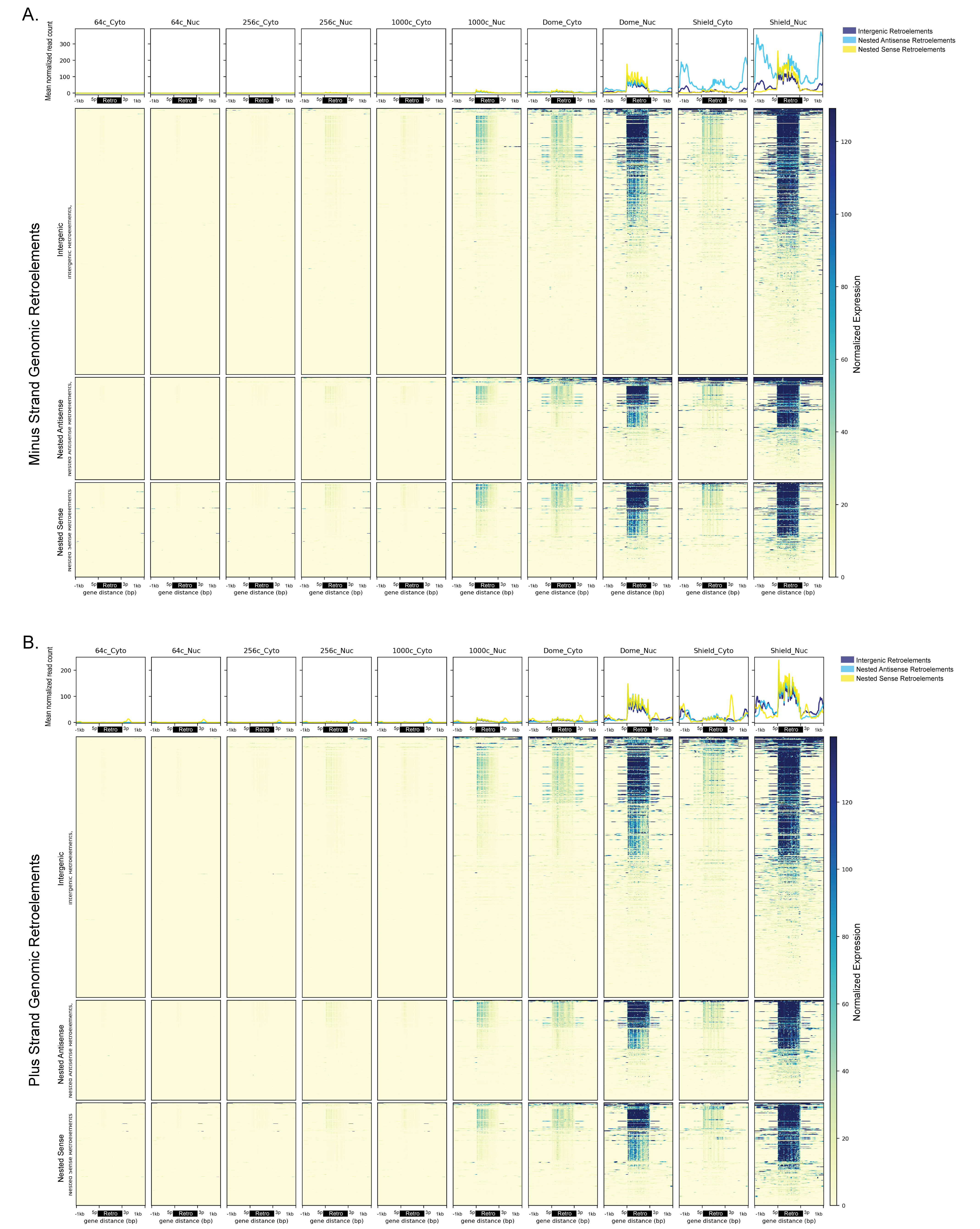

Supplement: Supplementary file 16 — Additional file 16. Metagene and heatmap analysis of intergenic, genic antisense and genic sense zebrafish retroelements from five different embryonic developmental stages (PRJNA599208) using unique and multi-mapped reads prepared using ribosomal RNA depletion protocols. Heatmaps of unique and multiply mapped reads from intergenic (second panel), genic antisense (third panel) and genic sense (bottom panel) zebrafish retroelement copies. Only the subset of full length retroelement copies with fewer than 10 mismatches or indels relative to their consensus sequence are included (see Additional File 17). Sample labels are indicated above each heatmap (from left to right- 64c_Cyto, 64c_Nuc, 256c_Cyto, 256c_Nuc, 1000c_Cyto, 1000c_Nuc, Dome_Cyto, Dome-Nuc, Shield_Cyto, Shield_Nuc where c denotes cells, Cyto denotes cytoplasmic and Nuc denotes nuclear fraction). Heatmap color scales represent the TMM normalized signal from RNA sequencing reads originating from the same strand as genomic negative strand (A) or genomic positive strand (B) retroelement copies. Metagene summary analyses (average normalized signal by relative position) for each sample and grouping are also presented (top panel, dark blue = average intergenic retroelement signal, light blue = average nested antisense retroelement signal, yellow = average nested sense retroelement signal). Please see Additional File 17 for genomic coordinates of retroelements included in these heatmaps. [file 13100_2022_287_MOESM16_ESM.tif]
